# Reduced microbial potential for the degradation of phenolic compounds in the rhizosphere of apple plantlets grown in soils affected by replant disease

**DOI:** 10.1186/s40793-019-0346-2

**Published:** 2019-11-07

**Authors:** Viviane Radl, Jana Barbro Winkler, Susanne Kublik, Luhua Yang, Traud Winkelmann, Gisle Vestergaard, Peter Schröder, Michael Schloter

**Affiliations:** 1 Research Unit Comparative Microbiome Analysis, Helmholtz Zentrum München, Munich, Germany; 2 Research Unit Environmental Simulations, Helmholtz Zentrum München, Munich, Germany; 3 Woody Plant and Propagation Physiology Section, Institute of Horticultural Production Systems, Leibniz Universität Hannover, Hanover, Germany; 4 Department of Health Technology, Section for Bioinformatics, Technical University of Denmark, Lyngby, Denmark

**Keywords:** Metagenome, Apple replant disease, *Malus domestica*, Microbiome, Rhizosphere

## Abstract

**Background:**

Apple replant disease (ARD) is a syndrome that occurs in areas where apple plants or closely related species have been previously cultivated. Even though ARD is a well-known phenomenon, which has been observed in different regions worldwide and occurs independent of the soil type, its causes still remain unclear.

**Results:**

As expected, the biomass of plants grown in replant soil was significantly lower compared to those grown in control (virgin) soil. A shotgun metagenome analysis showed a clear differentiation between the rhizosphere and bulk soil compartments independent from the soil used. However, significant differences associated with apple replant disease were only observed in the rhizosphere compartment, for which we detected changes in the abundance of major bacterial genera. Interestingly, reads assigned to Actinobacteria were significantly reduced in relative abundance in rhizosphere samples of the soil affected by replant disease. Even though reads assigned to pathogenic fungi were detected, their relative abundance was low and did not differ significantly between the two different soils. Differences in microbiome structure also resulted in shifts in functional pattern. We observed an increase in genes related to stress sensing in the rhizosphere of soils affected by replant disease, whereas genes linked to nutrient sensing and uptake dominated in control soils. Moreover, we observed a lower abundance of genes coding for enzymes which trigger the degradation of aromatic compounds in rhizosphere of soils affected by replant disease, which is probably connected with higher concentration of phenolic compounds, generally associated with disease progression.

**Conclusions:**

Our study shows, for the first time, how apple replanting affects soil functioning by altering the soil microbiome. Particularly, the decrease in the abundance of genes which code for enzymes catalyzing the degradation of aromatic compounds, observed in the rhizosphere of plants grown in soil affected by apple replant disease, is of interest. Apple rootstocks are known to synthetize many phenolic compounds, including defense related phytoalexins, which have been considered for long to be connected with the emergence of replant disease. The knowledge gained in this study might help to develop targeted strategies to overcome or at least reduce the effects of ARD symptoms.

## Background

Apple replant disease (ARD) is a phenomenon that has been mostly observed in areas where apple plants or closely related species of the family of *Rosaceae* have been repeatedly cultivated [1–3]. The disease is characterized by uneven growth throughout the orchard as well as the presence of shorter internodes and stunted growth of trees. Moreover, those trees often develop a smaller root system, with clear indications of decay or discoloring [4, 5]. ARD symptoms are most evident during the first 3 months following planting. In general, fruits of ARD affected trees are reduced in both, quality and quantity [1]. Even though ARD is a well-known phenomenon, which has been observed in different regions of the world and in a large variety of soil types [4, 6], its causes are still not well understood.

There is evidence for a relationship between ARD emergence and the accumulation of allelopathic compounds in the affected soils [7]. Even though allelochemicals might be degraded by soil microbes [8], in areas where apples have been cultivated for more than 20 years, phenolic compounds, e.g. benzoic acid and phlorizin, are present in significantly higher concentrations in the soil [9]. As showed by Nicola et al. [10] the concentration of total phenolics negatively correlated with the growth of the apple seedlings and were considered to be the cause of ARD [9]. However, a reduction of the typical symptoms of ARD was observed in studies where gamma-radiation, heating or broad spectrum fumigants had previously been applied to diseased soils, providing a strong evidence for the role of biotic factors on the emergence of the disease [11, 12].

In spite of the clear relationship between biotic factors and ARD emergence, up to now investigations failed to identify a disease-causing agent. Several studies pointed to the role of the nematode *Prathylencus penetrans*, a well-known plant parasite, on ARD development, while other studies associated the disease to the presence of pathogenic fungi and oomycetes, including *Cylindrocarpon*, *Rhizoctonia, Pythium* and *Phytophtora* [13, 14]. As postulated by Tewoldemedhin et al. [15], ARD is most probably caused by multiple biological agents, including many of the above cited organisms.

Even though pathogen complexes are certainly associated with ARD, they might not be the origin of the disease and play only a role as secondary infections at a time point when the plant is already weakened. Sensitive rootstocks showed an impaired response to ARD, with an accumulation of defense-related compounds in their roots, which can lead to initial root damage [16]. Moreover, it is possible that the changes in the soil microbiome caused by the establishment of apple orchards provide ideal conditions in which soils become permissive to pathogens. In fact, shifts on the bacterial community composition in replant soils have been frequently reported for both, bulk soil and rhizosphere compartment [17], such as changes in the abundance of *Burkholderia, Pseudomonas* [18] or Actinobacteria populations [19]. However, functional consequences of these shifts in the soil microbiome are not well understood. In the present study, we tested the hypothesis that ARD is facilitated by changes in the overall soil microbiome rather than by a simple increase of pathogen loads. The reduced abundance or the complete loss of keystone species and their associated functional traits might destabilize the system and facilitate the invasion by pathogens. Therefore, we conducted a greenhouse experiment employing the ARD susceptible rootstock genotype M26 grown in ARD soil and in a virgin soil, where no members of *Rosaceae* had been cultivated recently [20]. The aim of this study was to assess the soil microbiome and its major functional traits in bulk soil and in the rhizosphere of apple plants grown in healthy and ARD affected soil using a shotgun metagenomic approach.

## Results

### Identification of ARD symptoms

**Fig. 1:**
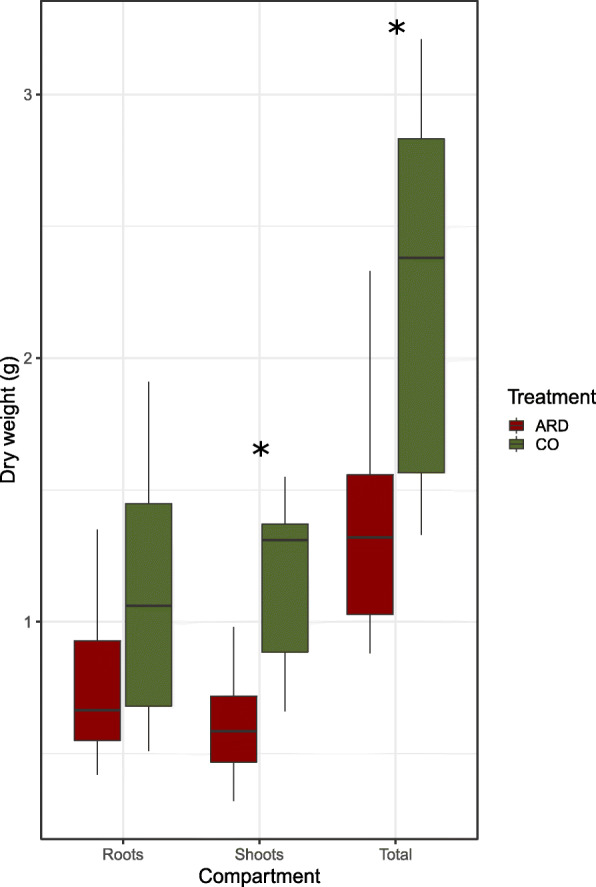
Dry mass of leaves, roots and total biomass 12 weeks after plantlets were transferred to pots containing virgin (CO = green) and apple replant soils (ARD = red), * = *p* < 0.05. *N* = 8 (Kruskal-Wallis-test)

We observed the typical disease symptoms on apple plants grown in replant soils. The roots were brownish and shorter with less fine roots and root hairs. Moreover, plants grown in ARD soil showed a significantly lower plant biomass (Kruskal-Wallis-Test, *p* < 0.05), which was due to a significantly decreased shoot dry mass (*p* < 0.05), whereas the differences for the root biomass were not significant (*p* > 0.05) (Fig. 1). We also observed a trend towards higher root: shoot ratio for plants grown in the diseased soil.

### Differences in the taxonomic composition of the microbiomes of ARD and CO rhizosphere soil

We obtained in average 3.2 million reads (±0.4) with read-lengths of 290 (±5) nucleotides (Additional file 1: Table S1) per sample. Nonpareil estimations of the metagenome average coverage of 3.5%. After all processing steps, sequences linked to bacterial genes were by far the most abundant in the metagenomes, comprising 60.02% (±1.7) of the reads obtained. Eukaryotes represented only 1.96% (±0.2) of the total reads, from which most were classified as fungi (58.7% ± 2.27). Sequences assigned to archaea and virus represented less than 0.55 and 0.07% of the total number of reads, respectively (Additional file 1: Table S2). Reads assigned to genes of bacteria from the phyla Proteobacteria, Actinobacteria, Bacteroidetes and Acidobacteria were most abundant in the rhizosphere metagenomes. Similar results were also obtained by the 16S rRNA amplicon sequencing approach (data not shown).

**Fig. 2:**
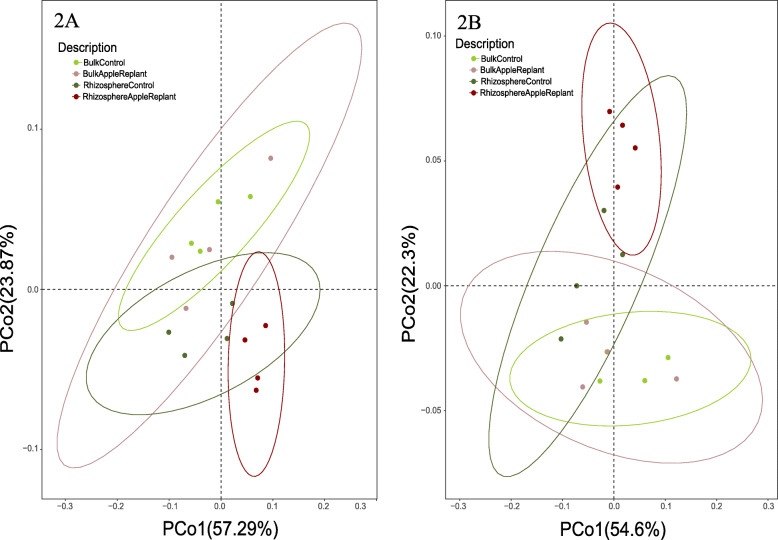
PCoA analysis generated from distance matrixes calculated using the Bray-Curtis method based on taxonomical (**a**) and functional (**b**) annotation of the metagenome reads

As expected, Bray-Curtis distance matrices based on the taxonomic annotations of the total metagenome reads revealed significant differences (Permanova, *p* < 0.05) between bulk soil and rhizosphere compartments, represented by the second axis of the PcoA plot (Fig. 2a). Moreover, in contrast to the bulk soil, for which ARD affected soil and CO soil could not be differentiated, we did observe significant differences (*p* < 0.05) in the composition of the metagenomes from the rhizosphere samples from apple plants grown in the CO and ARD affected soils. Therefore, further pairwise comparisons were only carried out for this compartment.

**Fig. 3:**
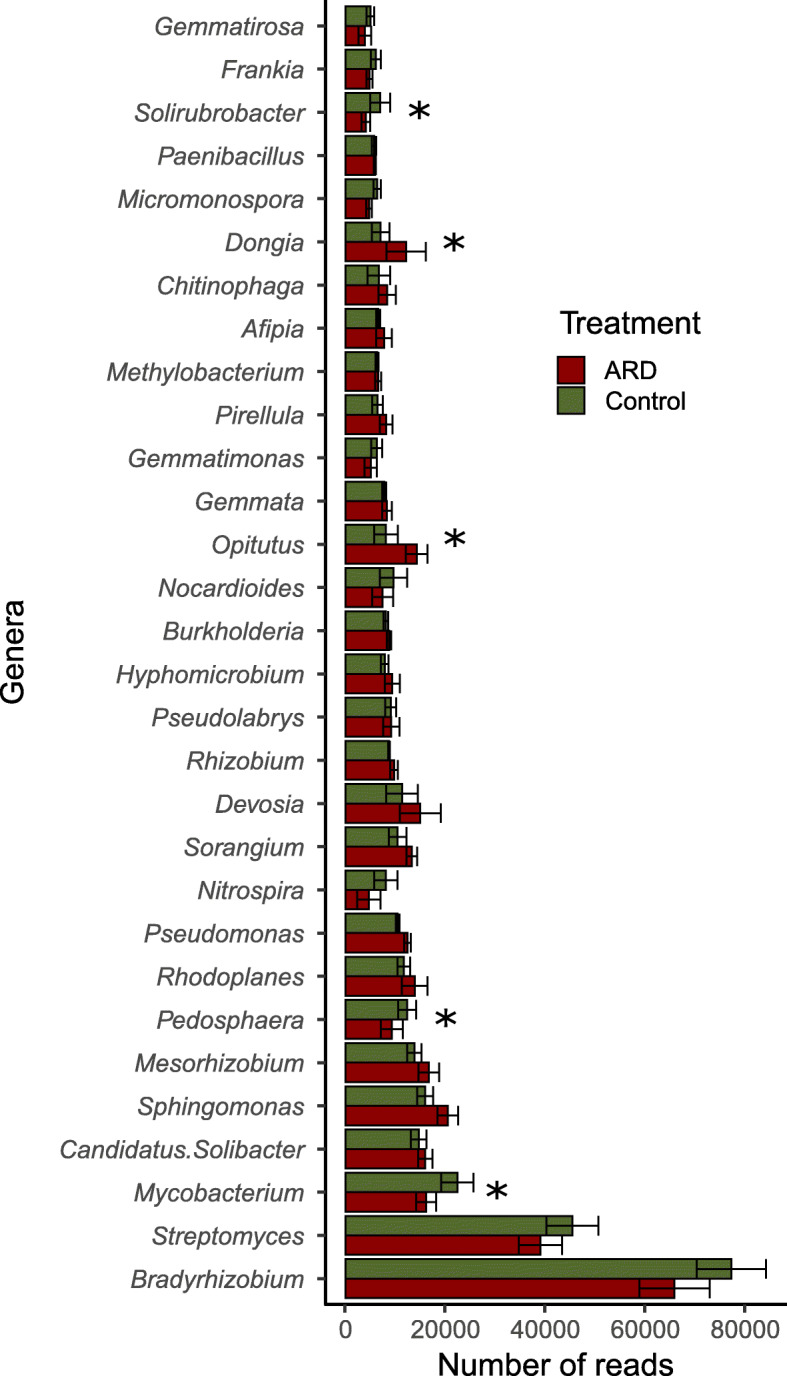
Relative number of reads (number of reads/total number of reads) assigned to the top 30 most abundant genera detected in the metagenome libraries. Rhizosphere samples collected 12 weeks after plantlets were transferred to pots containing virgin (CO = green) and apple replant soils (ARD = red), *N* = 4, * = *p* < 0.05 (DESeq2 analysis to test for differential gene abundance using negative binomial generalized linear models, p adjusted using the Bonferroni correction method for multiple pairwise comparisons)

Figure 3 shows the relative number of the reads assigned to the 30 most abundant genera identified in the metagenomes of ARD and CO treatments. *Bradyrhizobium*, *Streptomyces* and *Mycobacterium* were the most abundant genera detected in all the libraries.

We performed pairwise comparisons based on the taxonomic annotation of the reads using DESq2. Thereby, we identified the hits assigned to genera that were differently abundant (*p* < 0.05) in the rhizosphere of plants grown in ARD and CO soil (Additional file 1: Table S3). We detected 66 genera that were significantly higher in abundance in CO than in ARD, whereas only 37 were significantly more abundant in the ARD rhizosphere. Interestingly, significant differences between ARD affected and CO rhizosphere soils were mostly observed for genera of the phylum Actinobacteria. The strongest differences between the treatments (higher than 2 fold, Bonferoni corrected *p* < 0.05) were detected for *Friedmanniella*, *Terrabacter*, *Thermoleophilum* which were all lower in relative abundance in ARD affected soils and *Methylotenera,* which was lower in relative abundance in CO soil. These genera were not among the most dominant bacterial taxa found in our datasets. Nevertheless, significant differences were also found for dominant taxa. Lower number of reads assigned to the genera *Mycobacterium*, *Pedosphaera* and *Solirubrobacter* were detected in ARD affected compared to CO soil samples*.* In contrast, hits linked to the genera *Opitutus, Dongia*, *Novosphingobium* were more abundant in ARD affected than in CO soil samples.

We did not detect significant differences in the abundance of hits assigned to archaea, fungi or viruses. This applies to organisms usually described in the literature as causative agents detected in replant affected plants, such as *Rhizoctonia*, *Phytophtora* and *Ilyonectria*, which were detected in very low but similar relative abundances in the rhizosphere of ARD and CO plants. Moreover, we did not identify sequences assigned to *Pratylenchus penetrans* in our dataset.

### Functional potential of the microbial communities

After processing steps, 40% of the reads were assigned to KEGG functional categories. From those, 24.8, 5.8 and 5.6% belonged to the categories metabolism, genetic information processing and environmental information processing, respectively (Additional file 1: Table S1). In total, we obtained 5512 KEGG orthologues (KO). PCoA analysis based on function annotations of the reads showed similar patterns as observed for the taxonomic annotation (Fig. 2b). Therefore, further analyses were only performed for rhizosphere samples. DESeq2 analysis based on the overall data detected 51 KOs that were differently abundant (*p* < 0.05) in metagenomes from ARD affected and CO rhizosphere soil samples, however those were not significant after Bonferroni correction for multiple pairwise comparisons (Additional file 1: Table S5). Therefore, further analyses were carried out at category levels “metabolism of xenobiotics”, “terpenoids and polyketides metabolism” and “environmental information processing”. Functions were only further considered when KOs from complete or almost complete pathways showed the same response to the treatment.

### Potential for the degradation of aromatic compounds

We investigated genes related to the degradation of aromatic compounds as those compounds had previously been considered to play an important role in ARD (Nicola et al. 2016). NMF analyses were carried out to facilitate the detection of general patterns within CO and ARD metagenomes. We observed a lower abundance of reads assigned to genes related to the degradation of aromatic compounds (KEGG category “metabolism of xenobiotics”) in ARD affected compared to in CO rhizosphere soil samples (Additional file 1: Figure S1). From the 180 KOs detected for this category, 112 showed higher numbers of reads in CO, 5 of them showed more than 4 fold differences. Even though in most of the cases differences where not significant, the data shows a clear trend towards a lower potential to degrade aromatic compounds in the rhizosphere of plants grown in replant soil. This result was confirmed by DESeq2 analysis, which detected 13 differently abundant genes (Fig. 3a, *p* < 0.05). The majority of them are part of the benzoate or aminobenzoate degradation pathways (*namA, hcrA/hbaC bcrA/badF, acd,* and *badA/E*; Fig. 3A), as shown in Fig. 3b. We detected all genes related to the biphenyl degradation pathway (*bphA*, *B*, *C* and *D*) in our samples, however, those were low abundant and did not significantly differ between the libraries.

### Antagonist interactions: potential for antibiotic synthesis

**Fig. 4:**
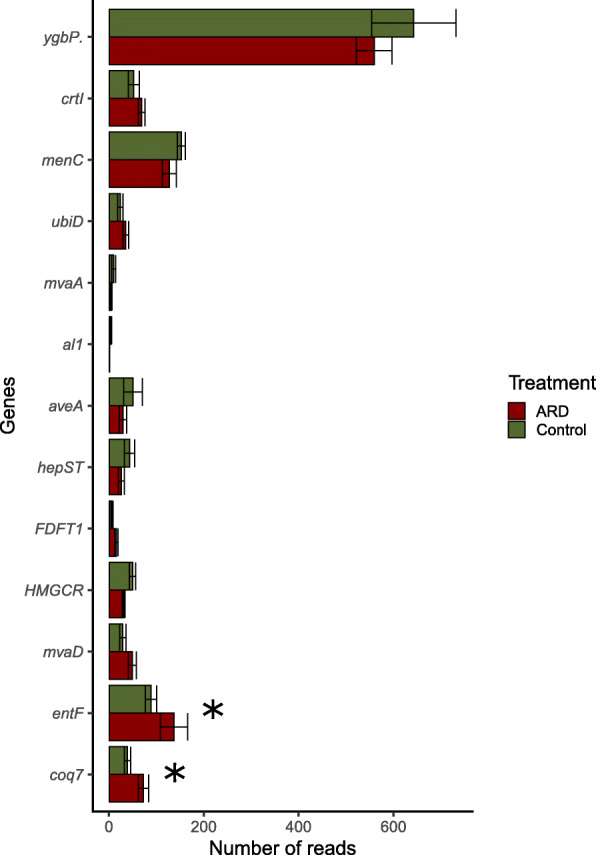
Relative number of reads (number of reads/total number of reads) assigned to genes involved in the synthesis and metabolism of terpenoids and polyketides. Rhizosphere samples collected 12 weeks after plantlets were transferred to pots containing virgin (CO = green) and apple replant soils (ARD = red), * = *p* < 0.05 (DESeq2 analysis to test for differential gene abundance using negative binomial generalized linear models, p adjusted using the Bonferroni correction method for multiple pairwise comparisons). Abbreviations: coq7 = ubiquinone biosynthesis monooxygenase, entF = enterobactin synthetase component F, HMGCR = hydroxymethylglutaryl-CoA reductase, FDFT1 = farnesyl-diphosphate farnesyltransferase, hepST = heptaprenyl diphosphate synthase, AVES = type I polyketide synthase, AL1 = phytoene desaturase, mvaA = hydroxymethylglutaryl-CoA reductase, UbiD = 3-octaprenyl-4-hydroxybenzoate carboxy-lyase, menC = O-succinylbenzoate synthase, crtI = phytoene desaturase, ygbP = 2-C-methyl-D-erythritol 4-phosphate cytidylyltransferase

**Fig. 5:**
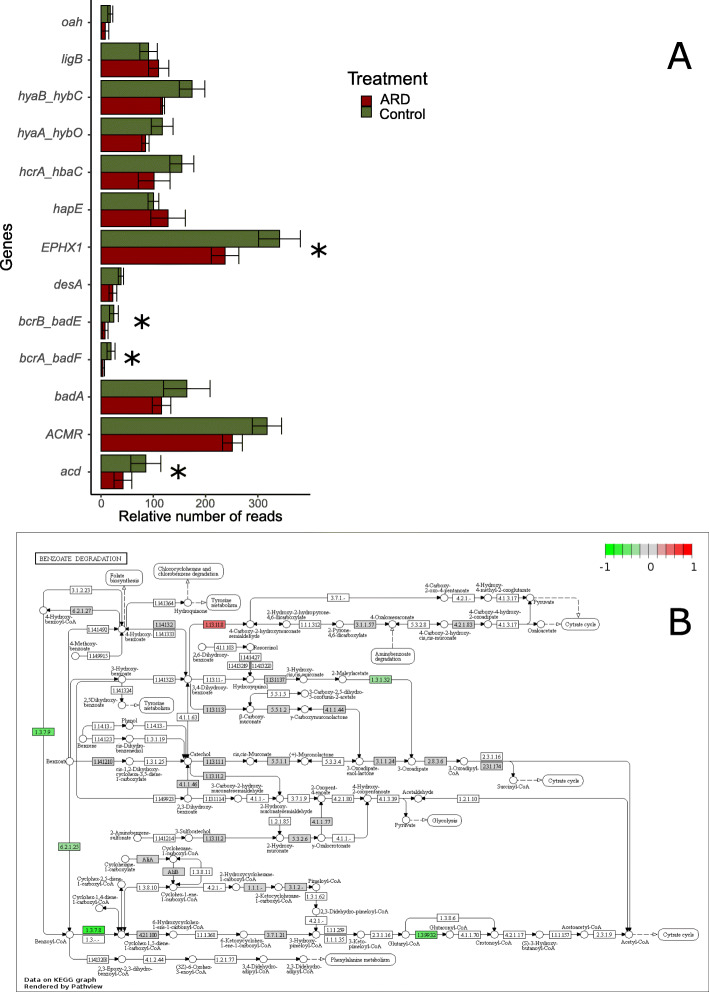
Relative number of reads (number of reads/total number of reads) assigned to genes involved in the degradation of aromatic compounds (category metabolism of xenobiotics). Rhizosphere samples collected 12 weeks after plantlets were transferred to pots containing virgin (CO = green) and apple replant soils (ARD = red), * = *p* < 0.05 (DESeq2 analysis to test for differential gene abundance using negative binomial generalized linear models, p adjusted using the Boferroni correction method for multiple pairwise comparisons). Pathview of the DESeq2 results for the category (red and green represent reads in higher or low abundances in ARD in comparison to CO. Abbreviation: EPHX = microsomal epoxide hydrolase, bcrA_badF = benzoyl-CoA reductase subunit (**a)**, acd = glutaryl-CoA dehydrogenase, hyaB_hybC = hydrogenase large subunit, bcrB_badE = benzoyl-CoA reductase subunit (**b)**, hcrA_hbaC = 4-hydroxybenzoyl-CoA reductase subunit alpha, hapE 4-hydroxyacetophenone monooxygenase, ACMR = anthraniloyl-CoA monooxygenase, badA = benzoate-CoA ligase, ligB = protocatechuate 4,5-dioxygenase beta chain, hyaA_hybO = hydrogenase small subunit, desA = syringate O-demethylase and oah = 6-oxo-cyclohex-1-ene-carbonyl-CoA hydrolase

Secondary metabolites, including polyketides and terpenoids, are highly relevant for interactions between microorganisms and have been associated with the disease suppression [21, 22]. Therefore, we also investigated the abundance patterns of genes related terpenoids and polyketides metabolism. We did not find a distinct pattern for the different soils (Additional file 1: Figure S2). However, using DESeq2 analysis 14 genes were detected that were differently abundant in CO and ARD affected rhizosphere soil samples (*p* < 0.05) (Fig. 4). Many of those genes were associated with the biosynthesis of ubiquinones. Genes coding for enzymes involved in the biosynthesis of ubiquinone (*coq7*), siderophore enterobactin synthase (*entF*) as well as the mevalonate-based synthesis of the terpenoid backbone (*mvaA* and *mvaD*) and the type I polyketide synthase related to the biosynthesis of avermectin (*aveA*), were detected in higher abundance in the rhizosphere of plants grown in ARD affected soils (Fig. 5). From those, only *coq7* and *entF* were significantly different after Bonferoni correction. We detected complete pathways for the synthesis of ubiquinone for eukaryotes (*coq2, coq3, coq6, coq5, coq7*) and prokaryotes (*ubiA*, *ubiD*, *ubiX*, *ubiB* 4, *ubiG*, *ubiH*, *ubiE*, *ubiF*). However, only for the first group differences between treatments were observed. Furthermore, we screened for the presence of complete pathways for the synthesis of antibiotics in the libraries. Full pathways of many lipopeptide antibiotics were detected in the libraries and are summarized in Additional file. We found a trend towards higher abundances of antibiotic synthetizing microbes in the rhizosphere of ARD affected soil.

### Potential interactions with the plant host

**Fig. 6:**
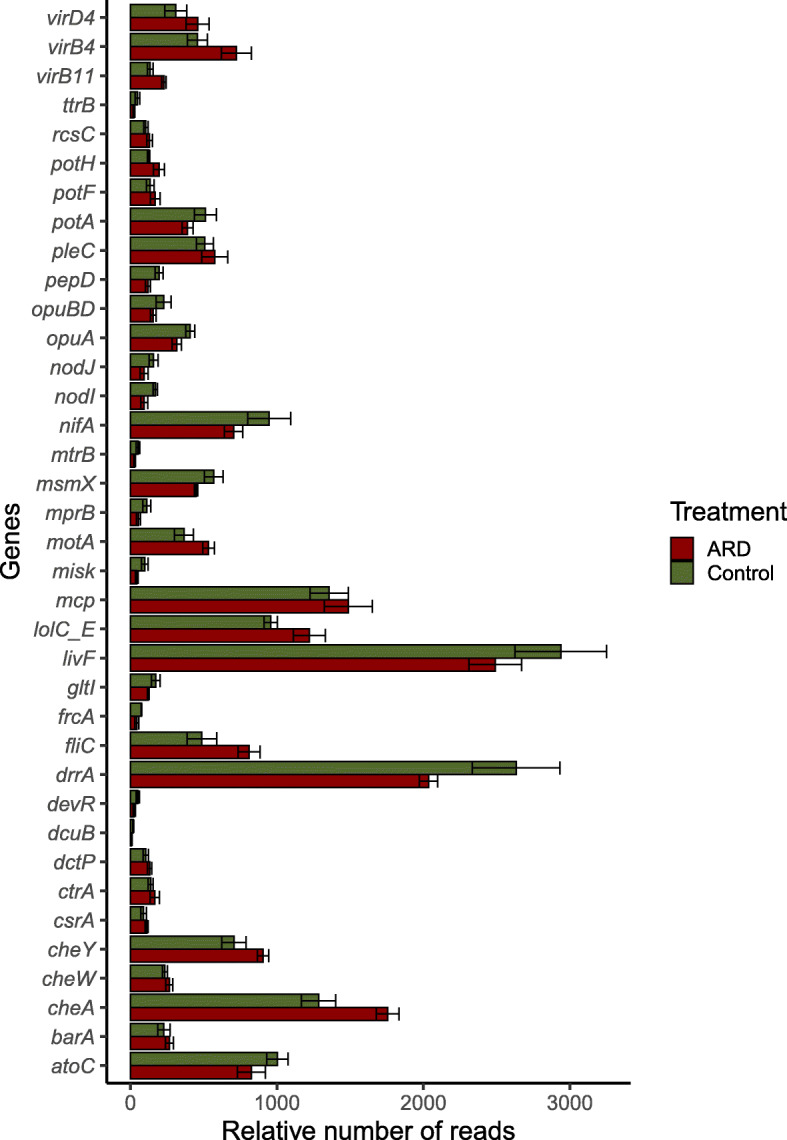
Relative number of reads (number of reads/total number of reads) assigned to genes from the category environmental information processing. Rhizosphere samples collected 12 weeks after plantlets were transferred to pots containing virgin (CO = green) and apple replant soils (ARD = red) (DESeq2 analysis to test for differential gene abundance using negative binomial generalized linear models, all genes showed *p* < 0.05 after adjusted using the Bonferroni correction method for multiple pairwise comparisons). Abbreviations: opuA = osmoprotectant transport system, virD4 = Type secretion system IV, potA = spermidine/putrescine transport system, potH = putrescine transport system, opuBD = osmoprotectant permease, livF = amino acid transport system, cheY = TCS chemotaxis, atoC = TCS NtrC family, gltI = glutamate/aspartate transport system, mcp = chemotaxis protein, potF = putrescine transport system, drrA = antibiotic transport system, dctP = C4-dicarboxylate-binding protein DctP, pleC = TCS sensor kinase, dcuB = anaerobic C4-dicarboxylate transporter DcuB, frcA = fructose transport system, ttrB = tetrathionate reductase subunit B, cheW = purine-binding chemotaxis protein, devR = TCS NarL family, nifA = regulatory protein, barA = TCS NarL family, ctrA = cell cycle response regulator CtrA, rcsC = TCS NarL family, csrA = carbon storage regulator, mtrB = TCS OmpR sensor kinase

Differential abundance analysis of genes assigned to the KEGG category “environmental information processing” showed differences in the rhizosphere microbiome of plants grown in CO and ARD affected soil (Fig. 6). Those are part of many KEGG modules relevant for the interactions between microbes and microbes as well as microbes and eukaryotic hosts. We detected 36 differently abundant genes. From those, 21 were assigned to two component systems, 12 to ABC transporters, 5 to bacterial chemotaxis, 4 to biofilm formation and 3 to bacterial secretion systems. In general, genes, which were more abundant in rhizosphere CO soil were related to nutrient sensing and uptake. This included two component systems for phosphate and nitrate/nitrite sensing, (OmpR and NarL), regulators of nitrogen fixation genes (*nifA*) as well as many sugar transport system, such as α-glucoside (*aglE, aglG* and *aglK*) and cellobiose (*cebE*, *cebF* and *msiK*). We detected an enrichment of quorum sensing regulators QseC in the rhizosphere of plants grown in ARD affected soils. Interestingly, also methyl-accepting chemotaxis proteins (MCPs) and genes involved in the transmission of sensory signals to the flagellar motors were more abundant in the microbiome of the rhizosphere of ARD affected soils. Stress sensing by the two component signal transduction system, RstB and ChvI were additionally more abundant in the rhizosphere samples of the ARD affected soil. We further identified a number of genes related to secretion systems of type I (full pathway), II, IV (full pathway) and VI. From those, *virB4* and *virB11* genes were significantly higher in relative abundance (*p* < 0.05 after Bonferroni correction) in the microbiome obtained from the rhizosphere of ARD affected soils (Additional file 1: Figure S3).

## Discussion

In the present study, we investigated the role of the soil microbiome and its functional properties as driver for apple replant disease. Thereby, we tested the hypothesis that ARD is not solely related to the increase of the abundance of pathogens but rather caused by changes in the assembly of the soil biota, particularly soil microbes, leading to changes in the soil functioning.

In fact, we found no evidence for an enrichment of pathogenic fungi or oomycetes in the replant soils. Yet, the presence of pathogenic nematodes cannot be excluded, as the methodology applied in our study was established to assess microbial communities and, hence, was inadequate for the detection of nematodes. However, plant-parasitic nematodes do not play a role on the occurrence of ARD in the soil of the site used in our experiment [23]. Overall, our data indicates that similar pathogen loads may lead to different plant growth patterns in virgin (CO) and ARD affected soils, with the last clearly causing disease symptoms. Though targeted methods, e.g. qPCR, for the quantification of identified agents infecting the damaged roots should be applied for a precise measurement of pathogen loads. Nevertheless, we assume that changes in the overall structure of the soil microbiome and its functional traits facilitated the colonization and establishment of pathogenic organisms at the plant-soil interface.

The composition of microbiome was very similar in the bulk soil of CO and ARD affected samples. Differences between CO and ARD affected soils became more evident in the rhizosphere compartment, which is in accordance to a recently published study carried out in apple nurseries located in replant and new planting sites in China [24].

We identified a few groups of bacteria that were more abundant in the rhizosphere of ARD affected soils, including *Opitutus*, *Dongia* and *Novosphingobium*. The prevalence of these bacteria was shown to be negatively correlated with the growth of apple plants [25, 26]. It is possible that they utilize plant-derived carbon in the rhizosphere of plants grown in replant soil. The only cultivated representative of the genus *Opitutus* isolated from soil was able to grow on plant-derived polysaccharides [27]. Similarly, members of the genus *Novosphingobium* were shown to harbor a number of mono- and dioxygenases responsible for the metabolism of several aromatic compounds [28]. These microbes might be better adapted to the conditions in replant soil (e.g. rhizodeposition) and, hence, have occupied niches inhabited by other microorganisms in the rhizosphere of healthy plants grown in control soil.

There is a great debate about mechanisms by which apple plants might alter microbial communities via root exudates and debris deposition. A recent study carried out by Leisso et al. [29] showed that tolerant and susceptible rootstock genotypes do differ in rhizodeposition. The authors found significantly higher concentrations of rutin in the rhizodeposits of the ARD susceptible rootstock M26, also used in the present study. This compound was shown to negatively affect bacterial growth [30]. Leisso et al. (2017) showed that total bacterial numbers, assessed by plate counting, were in general higher for deposits of tolerant rootstocks. Similarly, Weiß et al. [16] showed that M26 accumulates high amounts of phytoalexins, e.g. 3-hydroxy-5-methoxybiphenyl and aucuparin, in their roots when grown in ARD affected soils. Based on our metagenome data, it could be postulated that the presence of antimicrobial compounds in rhizodeposits of susceptible rootstocks increases stress and competition among microbes at the plant root interface, mainly in rhizosphere of plants grown in ARD affected soils. This is supported by the fact that we found increases in the relative abundances of genes related to environmental stress sensing, the synthesis of antibiotics, and a reduction of the abundance of genes from pathways associated with the metabolism of aromatic compounds in the rhizosphere of plants grown in ARD affected soils. Similar results were observed in studies on the effects of monoculture on soil health, for which changes taxonomic and functional composition are also accompanied by increases in disease incidence [31, 32]. Many of the genes or pathways detected in higher abundance in ARD, including Chv two-component regulatory system, type secretion system IV (regulated by Chv), genes related to cell motility (MPC) are frequently associated with the virulence of plant and human pathogens. However, the majority of these genes is also part of the machinery used by bacteria in interspecies competition [33]. While some species may be sensitive to changes related to ARD discussed above, others might engage in antibiotic synthesis, motility, predatory functions and biofilm formation in response to competition stress. Those mechanisms enhance the competitiveness of bacteria equipped with them and may be the cause of changes on microbial bacterial assembly often described in replant soil.

## Conclusions

Apple replant disease is a very complex and not completely understood phenomenon. With the development of many high throughput methods a lot of information was gained during recent years, which will help to elucidate the causes of ARD. The increasing number of data generated from affected areas worldwide can be used in the near future for metadata studies to define common patterns in this complex system. This might allow the development of efficient agricultural practices to overcome the problem of ARD. Here a central issue in the future will be how to drive the assembly of microbial communities in replant areas towards a disease suppressive state. Our data points to the relevance of microbes involved in the degradation of aromatic compounds in ARD affected soils, which needs to be proven for other areas and other settings worldwide.

## Material and methods

### Greenhouse experiment

In the present study we used top soil (0–20 cm) taken from an experimental apple nursery in Ellerhoop (Schleswig-Holstein, Germany). Since 2009 the area was separated in plots that were cultivated either with apples or maintained as grassland with defined species composition (Berliner Tiergarten grass mix: 50% *Lolium perenne*, 30% *Festuca rubra* rubra, 20% *Festuca rubra* communata). These soils are named throughout this manuscript as ARD and CO, respectively. The emergence of ARD at the plots used for apple cultivation was determined using bioassays (Yim et al. 2013) and details on soil type, soil texture and nutrient analyses (Mahnkopp et al., 2018) have been described elsewhere. The soil samples were stored at 4°C for approximately 1 week until the start of the experiment.

Four month old plantlets from the apple rootstock M26 were used in this experiment. They were propagated and rooted in vitro according to Yim et al. (2013). The peat substrate previously used for acclimatization of the clones was carefully removed from the roots and the plantlets were transferred to pots either containing 1.2 kg soil from ARD or CO plots. Plants were grown in greenhouse under the following conditions: 50% humidity, 12 h photoperiod and 22 °C and 15 °C day and night temperature. No fertilizer or plant protection agents were used during the experiment. Samples were taken 12 weeks after plants were transferred to the soil. This time point was selected based on 16S rRNA based barcode sequencing analysis carried out at different time points were (5, 10, 12 weeks). Here most pronounced differences in the rhizosphere microbiome structure between plants grown in CO and ARD affected soils were obtained after 12 weeks (data not shown). Therefore, metagenome analyses were performed for the last sampling point (12 weeks) using here bulk as well as rhizosphere samples for further analysis.

For each treatment 4 pots were used and treated as biological replicates for molecular biology analyses throughout. The plants were shaken to remove the soil which was not tightly adhered to the roots (defined here as bulk soil). The remaining soil was carefully separated from the roots by hand (defined here as rhizosphere). To avoid contamination gloves were changed after the collection of a sample. Rhizosphere and bulk soil samples used to investigate microbial communities were shock frozen and kept at − 80 °C until further analysis (below). ARD symptoms were asserted by visual inspection and the evaluation of root and shoot dry mass after 48 h drying at 105 °C.

### DNA extraction

Approximately 250 mg rhizosphere-and 500 mg of bulk soil were used for the extraction of nucleic acids following the protocol from Griffith et al. [34]. Briefly, samples were extracted using 10% hexadecyltrimethylammonium bromide and phenol: chloroform: isoamyl alcohol (25:24:1) using a homogenizer (Precellys, France) at 5500 strokes min-1. Quality and quantity of the DNA extracts were verified using an automated electrophoreses system (2100 Bioanalyzer, Agilent, USA) and Quant-iT PicoGreen dsDNA Assay Kit (Invitrogen, USA). We have blank sample and used as control for the metagenome analyses.

### Metagenome library preparation

1 μg DNA extract was sheared using the Covaris®E220 (Covaris, USA) using the following settings: incident power (W): 175, duty factor:2, cycles per burst: 200, treatment time: 35 s The fragments were treated by end repair A tailing. Size selection to 400–500 bp was performed with the Agencourt AMPure XP kit (Beckman Coulter, USA). Ligation of Illumina compatible adapters was done with the NEBNext Ultra II DNA Library Prep Kit for Illumina (New England Biolabs, USA) and the samples were multiplexed using NEBNext Multiplex Oligos for Illumina (NEB). Sequencing was performed on an Illumina® Miseq® (Illumina®) sequencing machine using the MiSeq® Reagent Kit v3 (600 cycles) (Illumina®) for paired end sequencing; details are given in in the Additional file 1: Table S1.

### Data analyses

After trimming, merging of forward and reverse reads using Adapterremoval v.2.1 (settings: 5′/3′- terminal minimum Phred quality = 15, minimum read length = 50) and removal of PhiX contaminants using Deconseq [35], reads were aligned against the KEGG database as of 2011 using Diamond [36]. Taxonomic annotation was performed using Kaiju and greedy mode allowing 5 substitutions [37] against the NCBI non-redundant protein blast database as of 18.01.2017. Read numbers of functional and taxonomical annotations were obtained using MEGAN v5.10.6 [38]. Following parameters were applied during MEGAN analysis: MinScore = 50.0, MaxExpected = 0.01, TopPercent = 10.0, MinSupport = 1, MinComplexity = 0. Hits with organism names containing words “environmental samples”, “unclassified” and “unidentified” were excluded from further analysis. For DESeq2 analyses no previous normalization steps were included, as DESeq2 includes the median of ratios normalization method, in which counts are divided by sample-specific size factors determined by median ratio of gene counts relative to geometric mean per gene. For other analysis, we normalized the data set using counts per million, in which counts are scaled by total number of reads. For better representation barplots show normalized reads scaled to 2 million reads.

For statistical analysis, the data obtained during bioinformatics analysis was submitted to multidimensional scaling analysis (PcoA) based on Bray-Curtis distance matrixes calculated from read tables from taxonomic and functional annotation using the R package ape version 5.1. Statistical significance of distances was determined by two-way PERMANOVA analysis (function adonis2 from R package “vegan”). We used non-negative matrix factorization analyses (NMF) to identify pattern in the metagenome data, as dimension-reduction methods focusing on dominating structures in the data might fail to depict alternative features and local behavior [39]. Visualization analyses based on KEGG annotation level 5 from the functional categories xenobiotic metabolism, metabolism of terpenoids and polyketides and environmental information processing were carried out using the NMF package. According to [40] DESeq2 is appropriate to evaluate data from high-throughput sequencing with low replication because it pools information across genes to estimate variance dispersion. Therefore, differential abundance analyses of the above mentioned datasets were carried out using the R package DESeq2 [41]. We only included sequences for which the sum of all reads was higher than 100 in the DESeq2 analyses. Problems related to multiple pairwise comparisons were considered as DESeq2 applies Bonferroni correction. Graphic visualization was performed with ggplot2 [42]. Pathview [43] was used to visualize DESeq2 results and search for changes in pathways rather than single genes.

The data from plant biomass was tested for normal distribution using the Shapiro-Wilk test. As the data was not normal distributed for all treatments, we applied the Kruskal-Wallis test.

## Supplementary information

**Additional file 1: Table S1.** Total number of reads obtained for the metagenome libraries. **Table S2.** Relative abundances of the reads assigned to different taxa. **Figure S1.** Nonpareil analysis to estimate the average cover of the metagenomes**.** Non-negative matrix factorization analysis of functional annotation (KEGG levels 5) of reads assigned to category xenobiotic metabolism **Table S3.** Results from DESeq2 analysis of rhizosphere samples based on taxonomic annotation of the data. Table shows pairwise comparisons between the treatments using control as base. Significant differences after Bonferoni correction (padj, * = < 0.05, ** = < 0.001) are highlighted in bold. **Table S4.** Functional categories annotation of reads. **Table S5.** Results from DESeq2 analysis of rhizosphere samples based on functional annotation of the overall data. Table shows pairwise comparisons between the treatments using control as base (only reads with *p* < 0.05) are shown. **Figure S2.** Non-negative matrix factorization analysis of functional annotation (KEGG levels 5) of reads assigned to the category metabolism of terpenoids and polyketides. **Figure S3.** a, b and c: Pathview showing the results of DESeq2 analysis of the category environmental information processing. Red and green represent reads detected in higher or lower abundance in ARD compared with CO, respectively.

## Data Availability

Datasets generated/analyzed during this study is available in the GenBank repository under the Bioproject accession number PRJNA532820.
