# Supplementary material for: Reduced microbial potential for the degradation of phenolic compounds in the rhizosphere of apple plantlets grown in soils affected by replant disease
Source: Environ Microbiome. 2019 Nov 7;14:8. doi: 10.1186/s40793-019-0346-2 (PMC8204438; doi:10.1186/s40793-019-0346-2)
Supplement: Supplementary file 1 — Additional file 1: Table S1. Total number of reads obtained for the metagenome libraries. Table S2. Relative abundances of the reads assigned to different taxa. Figure S1. Nonpareil analysis to estimate the average cover of the metagenomes. Non-negative matrix factorization analysis of functional annotation (KEGG levels 5) of reads assigned to category xenobiotic metabolism Table S3. Results from DESeq2 analysis of rhizosphere samples based on taxonomic annotation of the data. Table shows pairwise comparisons between the treatments using control as base. Significant differences after Bonferoni correction (padj, * = < 0.05, ** = < 0.001) are highlighted in bold. Table S4. Functional categories annotation of reads. Table S5. Results from DESeq2 analysis of rhizosphere samples based on functional annotation of the overall data. Table shows pairwise comparisons between the treatments using control as base (only reads with p < 0.05) are shown. Figure S2. Non-negative matrix factorization analysis of functional annotation (KEGG levels 5) of reads assigned to the category metabolism of terpenoids and polyketides. Figure S3. a, b and c: Pathview showing the results of DESeq2 analysis of the category environmental information processing. Red and green represent reads detected in higher or lower abundance in ARD compared with CO, respectively. [file 40793_2019_346_MOESM1_ESM.docx]

Additional file 1

Table S1: Total number of reads obtained for the metagenome libraries

| **Sample** | **Treatment** | **Compartment** | **Number of reads** | **Average length** |
| --- | --- | --- | --- | --- |
| ARD_1 | Apple replant | Bulk soil | 2778492 | 301 |
| ARD_2 | Apple replant | Bulk soil | 3768662 | 291 |
| ARD_3 | Apple replant | Bulk soil | 3437178 | 283 |
| ARD_4 | Apple replant | Bulk soil | 3635632 | 289 |
| CO_1 | Control | Bulk soil | 2697182 | 290 |
| CO_2 | Control | Bulk soil | 3743098 | 294 |
| CO_3 | Control | Bulk soil | 2952802 | 293 |
| CO_4 | Control | Bulk soil | 3438766 | 287 |
| ARD_1 | Apple replant | Rhizosphere | 3003312 | 294 |
| ARD_2 | Apple replant | Rhizosphere | 2989936 | 291 |
| ARD_3 | Apple replant | Rhizosphere | 3071916 | 292 |
| ARD_4 | Apple replant | Rhizosphere | 3071710 | 287 |
| CO_1 | Control | Rhizosphere | 3165500 | 283 |
| CO_2 | Control | Rhizosphere | 3760708 | 290 |
| CO_3 | Control | Rhizosphere | 3417148 | 288 |
| CO_4 | Control | Rhizosphere | 4159210 | 284 |
|  |  |  |  |  |
|  |  | **Minimum** | 2697182 | 283 |
|  |  | **Maximum** | 4159210 | 301 |
|  |  | **Average** | 3318203 | 290 |
|  |  | **Std. deviation** | 402175 | 5 |

Table S2: Relative abundances of the reads assigned to different taxa

|  | **Archaea** | **Bacteria** | **Eukaryotes** | | **Virus** | **Unassigned** |
| --- | --- | --- | --- | --- | --- | --- |
|  |  |  | **All** | **Fungi** |  |  |
| **CO_1_Bulk** | 0.74 | 61.39 | 2.04 | 1.24 | 0.07 | 35.39 |
| **CO_2_Bulk** | 0.81 | 57.63 | 1.73 | 1.07 | 0.07 | 39.48 |
| **CO_3_Bulk** | 0.74 | 62.42 | 2.21 | 1.32 | 0.08 | 34.14 |
| **CO_4_Bulk** | 0.63 | 60.01 | 1.81 | 1.09 | 0.06 | 37.17 |
| **ARD_1_Bulk** | 1.17 | 56.20 | 1.58 | 0.94 | 0.05 | 40.73 |
| **ARD_2_Bulk** | 0.73 | 61.15 | 1.88 | 1.15 | 0.06 | 35.83 |
| **ARD_3_Bulk** | 0.66 | 58.26 | 1.65 | 0.99 | 0.05 | 39.09 |
| **ARD_4_Bulk** | 0.55 | 61.52 | 2.14 | 1.27 | 0.07 | 35.33 |
| **CO_1_Rhizo** | 0.38 | 59.93 | 2.16 | 1.25 | 0.07 | 37.11 |
| **CO_2_Rhizo** | 0.40 | 61.48 | 2.13 | 1.24 | 0.07 | 35.56 |
| **CO_3_Rhizo** | 0.36 | 59.06 | 1.94 | 1.13 | 0.06 | 38.26 |
| **CO_4_Rhizo** | 0.51 | 58.64 | 1.79 | 1.06 | 0.06 | 38.68 |
| **ARD_1_Rhizo** | 0.28 | 61.17 | 2.25 | 1.23 | 0.07 | 35.90 |
| **ARD_2_Rhizo** | 0.34 | 58.48 | 2.07 | 1.08 | 0.06 | 38.72 |
| **ARD_3_Rhizo** | 0.32 | 61.62 | 2.11 | 1.26 | 0.07 | 35.54 |
| **ARD_4_Rhizo** | 0.25 | 61.44 | 1.83 | 1.07 | 0.06 | 36.10 |
|  |  |  |  |  |  |  |
| **Average** | 0.55 | 60.02 | 1.96 | 1.15 | 0.07 | 37.06 |
| **Std. Deviation** | 0.24 | 1.73 | 0.20 | 0.11 | 0.01 | 1.82 |

Figure S1: Nonpareil analysis to estimate the average cover of the metagenomes.


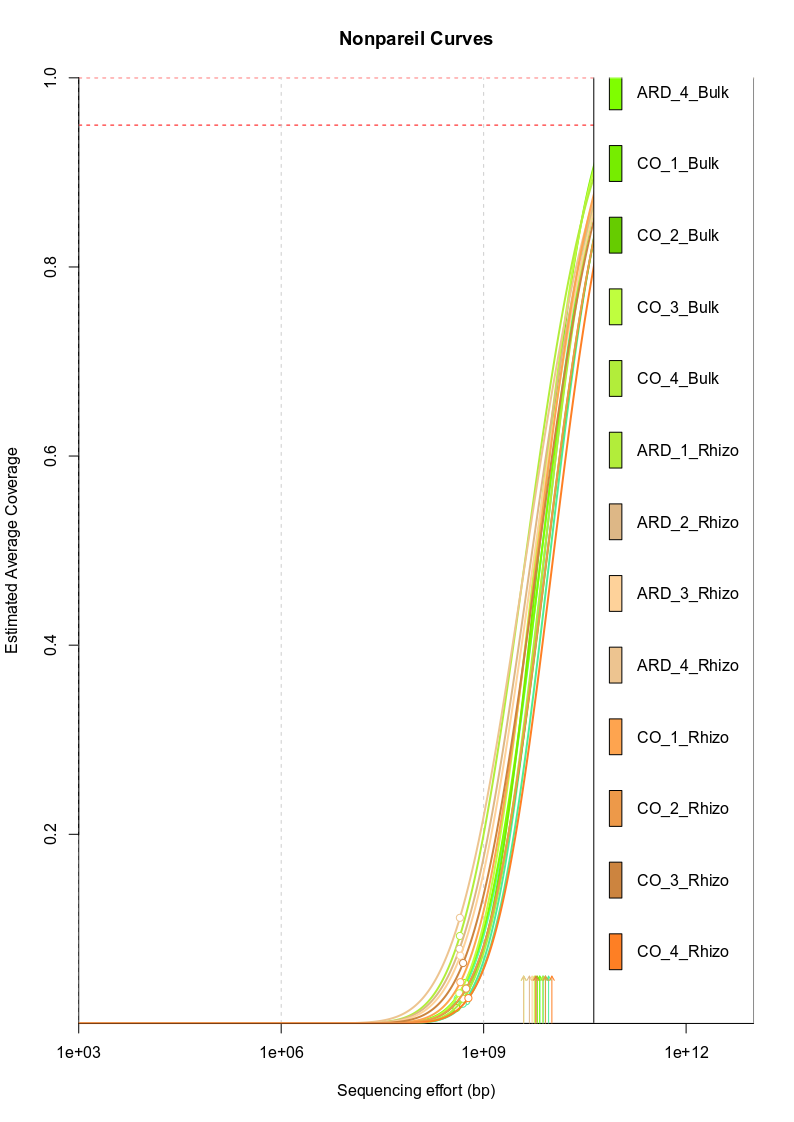


Table S3: Results from DESeq2 analysis of rhizosphere samples based on taxonomic annotation of the data. Table shows pairwise comparisons between the treatments using control as base. Significant differences after Bonferoni correction (padj, * = <0.05, ** = < 0.001) are highlighted in bold .

| ***Taxa*** | ***baseMean*** | ***log2FoldChange*** | ***lfcSE*** | ***stat*** | ***pvalue*** | ***Padj*** |
| --- | --- | --- | --- | --- | --- | --- |
| Bradyrhizobium | 597669.1 | -0.2585 | 0.11058 | -2.3377 | 0.019403 | 0.12 |
| Streptomyces | 352544.6 | -0.24822 | 0.112091 | -2.21449 | 0.026795 | 0.15 |
| **Mycobacterium** | **161313** | **-0.4569** | **0.11992** | **-3.81** | **0.00014** | **0.00** |
| Sphingomonas | 151415.7 | 0.25984 | 0.106847 | 2.431904 | 0.01502 | 0.10 |
| Candidatus Solibacter | 127601 | 0.050045 | 0.106887 | 0.468208 | 0.639636 | 0.82 |
| Mesorhizobium | 126783.1 | 0.179867 | 0.112495 | 1.598902 | 0.109842 | 0.33 |
| Devosia | 109247.2 | 0.249027 | 0.147503 | 1.688453 | 0.091324 | 0.29 |
| Rhodoplanes | 106284.5 | 0.146952 | 0.124049 | 1.184636 | 0.236161 | 0.50 |
| Sorangium | 98497.07 | 0.243678 | 0.114099 | 2.135698 | 0.032704 | 0.17 |
| Pseudomonas | 95040.58 | 0.170291 | 0.089892 | 1.894388 | 0.058174 | 0.22 |
| **Opitutus** | **92539.4** | **0.58269** | **0.13754** | **4.23762** | **2.26E-05** | **0.00** |
| **Pedosphaera** | **90418** | **-0.3898** | **0.13211** | **-2.9509** | **0.00317** | **0.04** |
| **Dongia** | **79365.6** | **0.50775** | **0.15112** | **3.36191** | **0.00077** | **0.01** |
| Rhizobium | 76762.35 | 0.087225 | 0.096089 | 0.907753 | 0.364009 | 0.63 |
| Pseudolabrys | 76029.13 | -0.04246 | 0.124056 | -0.34226 | 0.732157 | 0.87 |
| Hyphomicrobium | 71795.28 | 0.156334 | 0.119065 | 1.313021 | 0.189176 | 0.43 |
| Nocardioides | 71301.97 | -0.30289 | 0.150963 | -2.00678 | 0.044773 | 0.20 |
| Burkholderia | 69986.78 | 0.040059 | 0.09229 | 0.434056 | 0.664248 | 0.83 |
| Gemmata | 66834.82 | 0.032263 | 0.105537 | 0.305708 | 0.759827 | 0.88 |
| Chitinophaga | 62184.73 | 0.19454 | 0.144122 | 1.349894 | 0.17705 | 0.42 |
| Pirellula | 60401.71 | 0.22592 | 0.119761 | 1.886448 | 0.059235 | 0.23 |
| Afipia | 59334.94 | 0.148845 | 0.121854 | 1.221503 | 0.221895 | 0.48 |
| Nitrospira | 53716.66 | -0.32387 | 0.159819 | -2.04061 | 0.04129 | 0.19 |
| Methylobacterium | 53677.43 | -0.01568 | 0.10055 | -0.15597 | 0.876055 | 0.95 |
| **Novosphingobium** | **52280.6** | **0.53885** | **0.10069** | **5.35169** | **8.71E-08** | **0.00** |
| Niastella | 50619.25 | 0.149345 | 0.152381 | 0.980133 | 0.32702 | 0.59 |
| Mucilaginibacter | 48896.62 | 0.214621 | 0.148996 | 1.440576 | 0.149705 | 0.38 |
| Paenibacillus | 48291.53 | -0.02536 | 0.090618 | -0.27988 | 0.779569 | 0.89 |
| Chryseolinea | 47528.66 | 0.172555 | 0.147769 | 1.167791 | 0.242891 | 0.50 |
| Gemmatimonas | 47506.86 | -0.29255 | 0.135989 | -2.15137 | 0.031447 | 0.16 |
| Minicystis | 46660.34 | 0.139548 | 0.115661 | 1.206529 | 0.227614 | 0.49 |
| **Micromonospora** | **46504.8** | **-0.4237** | **0.1147** | **-3.6946** | **0.00022** | **0.01** |
| **Caulobacter** | **46440.6** | **0.3147** | **0.10466** | **3.00695** | **0.00264** | **0.03** |
| **Solirubrobacter** | **46320.6** | **-0.6007** | **0.14734** | **-4.0793** | **4.52E-05** | **0.00** |
| Frankia | 45711.69 | -0.33742 | 0.122673 | -2.75061 | 0.005949 | 0.06 |
| **Phenylobacterium** | **45371.6** | **0.37431** | **0.11524** | **3.24825** | **0.00116** | **0.02** |
| Variovorax | 43745.95 | -0.11885 | 0.124691 | -0.95313 | 0.340527 | 0.61 |
| Nocardia | 42897.39 | -0.29253 | 0.114415 | -2.55677 | 0.010565 | 0.08 |
| Pedobacter | 42637.06 | 0.091084 | 0.140433 | 0.648598 | 0.516599 | 0.74 |
| Chondromyces | 41812.88 | 0.225148 | 0.111392 | 2.021232 | 0.043256 | 0.19 |
| Planctomyces | 41691.49 | -0.01806 | 0.096337 | -0.18752 | 0.851255 | 0.93 |
| **Chthoniobacter** | **40426.6** | **-0.3058** | **0.1071** | **-2.8549** | **0.00431** | **0.05** |
| **Singulisphaera** | **40297.3** | **-0.2816** | **0.09332** | **-3.0172** | **0.00255** | **0.03** |
| Flavihumibacter | 38476.11 | 0.107703 | 0.14867 | 0.724462 | 0.468782 | 0.71 |
| Flavobacterium | 38347.9 | 0.078359 | 0.140984 | 0.555801 | 0.578347 | 0.79 |
| Bacillus | 37718.82 | -0.14074 | 0.096182 | -1.46329 | 0.143387 | 0.38 |
| Gemmatirosa | 37087.31 | -0.32476 | 0.143276 | -2.26699 | 0.023391 | 0.14 |
| Rhodopseudomonas | 36957.07 | 0.07538 | 0.116417 | 0.647501 | 0.517308 | 0.74 |
| Bosea | 36582.82 | 0.079027 | 0.104987 | 0.75273 | 0.451612 | 0.70 |
| **Bryobacter** | **36176.9** | **0.42129** | **0.10745** | **3.92075** | **8.83E-05** | **0.00** |
| **Sandaracinus** | **34714.3** | **0.47787** | **0.11803** | **4.04883** | **5.15E-05** | **0.00** |
| Rhodopirellula | 33954.7 | 0.093025 | 0.101301 | 0.9183 | 0.358462 | 0.63 |
| **Pseudonocardia** | **33923.3** | **-0.5699** | **0.1319** | **-4.3214** | **1.55E-05** | **0.00** |
| **Sphingobium** | **33063.6** | **0.34487** | **0.09869** | **3.4945** | **0.00047** | **0.01** |
| Microvirga | 32837.88 | -0.18506 | 0.101252 | -1.8277 | 0.067594 | 0.24 |
| Labilithrix | 32696.92 | 0.120778 | 0.112696 | 1.071722 | 0.283845 | 0.55 |
| Flavisolibacter | 32571.63 | 0.098217 | 0.154831 | 0.63437 | 0.52584 | 0.75 |
| Bauldia | 32279.51 | 0.36088 | 0.144596 | 2.496215 | 0.012553 | 0.09 |
| **Rhodococcus** | **31967** | **-0.3854** | **0.11868** | **-3.247** | **0.00117** | **0.02** |
| Paraburkholderia | 31709.69 | -0.01939 | 0.103668 | -0.18702 | 0.851647 | 0.93 |
| Schlesneria | 30605.7 | 0.050196 | 0.099153 | 0.506247 | 0.612683 | 0.81 |
| Zavarzinella | 30569.64 | -0.23496 | 0.097161 | -2.41825 | 0.015595 | 0.10 |
| Conexibacter | 30399.58 | -0.07863 | 0.164043 | -0.47939 | 0.631658 | 0.81 |
| **Actinoplanes** | **30302.7** | **-0.4829** | **0.13728** | **-3.5179** | **0.00043** | **0.01** |
| **Amycolatopsis** | **28697.7** | **-0.3771** | **0.11728** | **-3.2154** | **0.0013** | **0.02** |
| Asticcacaulis | 28351.68 | 0.260304 | 0.140869 | 1.847977 | 0.064606 | 0.23 |
| Haliangium | 28312.29 | 0.016751 | 0.116849 | 0.143354 | 0.88601 | 0.95 |
| Verrucomicrobium | 28291.08 | 0.034114 | 0.090235 | 0.378053 | 0.705392 | 0.85 |
| Myxococcus | 27837.97 | -0.00229 | 0.103307 | -0.02216 | 0.982319 | 0.99 |
| **Rhizomicrobium** | **27347.6** | **0.51495** | **0.13651** | **3.77297** | **0.00016** | **0.00** |
| **Methyloceanibacter** | **27011.7** | **-0.7754** | **0.1492** | **-5.203** | **1.96E-07** | **0.00** |
| Anaeromyxobacter | 26419.43 | -0.10787 | 0.09738 | -1.10776 | 0.267966 | 0.53 |
| Kaistia | 25977.6 | 0.348854 | 0.130801 | 2.667213 | 0.007648 | 0.07 |
| **Arthrobacter** | **25746.3** | **-0.6012** | **0.12915** | **-4.6559** | **3.23E-06** | **0.00** |
| Microbacterium | 25459.38 | -0.26858 | 0.119747 | -2.24292 | 0.024902 | 0.14 |
| Desulfovibrio | 25282.16 | -0.04907 | 0.087789 | -0.55891 | 0.576221 | 0.78 |
| Azospirillum | 25206.43 | 0.155486 | 0.096951 | 1.603768 | 0.108765 | 0.32 |
| Massilia | 25108.47 | 0.158993 | 0.093467 | 1.701058 | 0.088932 | 0.29 |
| **Rhodanobacter** | **24704** | **0.39774** | **0.13646** | **2.91508** | **0.00356** | **0.04** |
| Candidatus Koribacter | 24447.65 | 0.026658 | 0.125262 | 0.212821 | 0.831467 | 0.92 |
| Blastopirellula | 24295.25 | 0.233005 | 0.118416 | 1.967703 | 0.049102 | 0.21 |
| Niabella | 24194.9 | 0.099423 | 0.164423 | 0.60478 | 0.545325 | 0.76 |
| Lysobacter | 23483.99 | 0.123037 | 0.112146 | 1.09712 | 0.272589 | 0.53 |
| Hyalangium | 23039.79 | -0.1327 | 0.150003 | -0.88466 | 0.376338 | 0.64 |
| Leptolyngbya | 22433.27 | 0.002 | 0.100224 | 0.019955 | 0.984079 | 0.99 |
| Hassallia | 21989.47 | 0.1943 | 0.144072 | 1.348702 | 0.177433 | 0.42 |
| Clostridium | 21688.34 | -0.03046 | 0.096059 | -0.31709 | 0.751177 | 0.88 |
| Nitrobacter | 21314.7 | 0.109744 | 0.124594 | 0.880816 | 0.378417 | 0.64 |
| Actinomadura | 21044.38 | -0.33656 | 0.126229 | -2.66638 | 0.007667 | 0.07 |
| Cupriavidus | 20993.41 | -0.08122 | 0.095994 | -0.84612 | 0.397487 | 0.65 |
| **Hyphomonas** | **20714.3** | **0.5529** | **0.12911** | **4.28305** | **1.84E-05** | **0.00** |
| **Sphingopyxis** | **20410.3** | **0.31993** | **0.10414** | **3.07217** | **0.00213** | **0.03** |
| **Steroidobacter** | **19585.9** | **0.50403** | **0.13163** | **3.82974** | **0.00013** | **0.00** |
| Hymenobacter | 19428.1 | 0.032885 | 0.10865 | 0.302667 | 0.762144 | 0.88 |
| **Brevundimonas** | **19410** | **0.33763** | **0.11874** | **2.84352** | **0.00446** | **0.05** |
| Archangium | 19259.65 | -0.10272 | 0.103959 | -0.98811 | 0.323098 | 0.59 |
| Deinococcus | 18992.16 | -0.11435 | 0.087912 | -1.30077 | 0.193338 | 0.44 |
| Bordetella | 18692.11 | 0.146116 | 0.105197 | 1.388974 | 0.164841 | 0.41 |
| Parafilimonas | 18408.89 | -0.04957 | 0.153972 | -0.32194 | 0.747497 | 0.88 |
| Dyadobacter | 18276.8 | 0.086935 | 0.140411 | 0.619156 | 0.535814 | 0.75 |
| Terriglobus | 17992.66 | 0.039024 | 0.095711 | 0.407729 | 0.683473 | 0.84 |
| Spirosoma | 17988.68 | 0.058877 | 0.128998 | 0.456422 | 0.648087 | 0.82 |
| Bacteroides | 17738.44 | 0.068706 | 0.117762 | 0.583437 | 0.559599 | 0.77 |
| Stigmatella | 17645.67 | -0.0917 | 0.118092 | -0.77651 | 0.437446 | 0.69 |
| Xanthomonas | 17445.71 | 0.193038 | 0.104356 | 1.849813 | 0.06434 | 0.23 |
| Geobacter | 17202.87 | -0.12581 | 0.115651 | -1.08784 | 0.276666 | 0.54 |
| **Cellulomonas** | **16881.4** | **-0.415** | **0.12852** | **-3.2295** | **0.00124** | **0.02** |
| **Candidatus Entotheonella** | **16865.8** | **-0.4414** | **0.12686** | **-3.4795** | **0.0005** | **0.01** |
| Nannocystis | 16860.59 | 0.246592 | 0.123277 | 2.000346 | 0.045463 | 0.20 |
| Ktedonobacter | 16858.61 | -0.11285 | 0.096934 | -1.1642 | 0.244343 | 0.51 |
| Gimesia | 16655.82 | 0.035878 | 0.09913 | 0.361932 | 0.717403 | 0.86 |
| Pyrinomonas | 16601.16 | -0.40592 | 0.164489 | -2.47643 | 0.01327 | 0.10 |
| Nitrosomonas | 16428.7 | 0.115127 | 0.098604 | 1.167569 | 0.242981 | 0.50 |
| Segetibacter | 16392.26 | 0.035547 | 0.149997 | 0.236988 | 0.812666 | 0.91 |
| **Ilumatobacter** | **16264.9** | **-0.4935** | **0.14334** | **-3.4441** | **0.00057** | **0.01** |
| **Kouleothrix** | **16199.4** | **-0.4954** | **0.0983** | **-5.0398** | **4.66E-07** | **0.00** |
| Rhodovulum | 16175.91 | 0.101485 | 0.113116 | 0.897181 | 0.369622 | 0.63 |
| Roseomonas | 15950.9 | -0.01743 | 0.103011 | -0.16924 | 0.86561 | 0.94 |
| **Nocardiopsis** | **15622.1** | **-0.3287** | **0.11018** | **-2.983** | **0.00285** | **0.03** |
| **Nonomuraea** | **15607.5** | **-0.4206** | **0.12285** | **-3.4241** | **0.00062** | **0.01** |
| Magnetospirillum | 15518.17 | 0.187941 | 0.093148 | 2.01767 | 0.043626 | 0.19 |
| Candidatus Accumulibacter | 15377.02 | 0.002849 | 0.090345 | 0.031538 | 0.97484 | 0.99 |
| Planctomicrobium | 15228.81 | 0.027168 | 0.10015 | 0.271278 | 0.786177 | 0.90 |
| Legionella | 15121.79 | 0.154358 | 0.106915 | 1.443745 | 0.148811 | 0.38 |
| Granulicella | 14936.06 | 0.018419 | 0.107872 | 0.170749 | 0.864421 | 0.94 |
| **Geodermatophilus** | **14912.8** | **-0.5066** | **0.13434** | **-3.7719** | **0.00016** | **0.00** |
| Methylibium | 14737.45 | 0.121648 | 0.134837 | 0.902193 | 0.366955 | 0.63 |
| Acidovorax | 14358.52 | 0.024242 | 0.092434 | 0.262267 | 0.793115 | 0.90 |
| **Gaiella** | **14335.8** | **-0.5766** | **0.14865** | **-4.1504** | **3.32E-05** | **0.00** |
| Halomonas | 14157.33 | 0.120904 | 0.102677 | 1.177522 | 0.238987 | 0.50 |
| Dokdonella | 14110.37 | 0.251467 | 0.141236 | 1.780593 | 0.074979 | 0.26 |
| Chryseobacterium | 13941.63 | 0.03042 | 0.139276 | 0.218414 | 0.827107 | 0.92 |
| Pseudoxanthomonas | 13871.71 | 0.304317 | 0.116079 | 2.621685 | 0.00875 | 0.07 |
| Algoriphagus | 13850.48 | 0.082615 | 0.127682 | 0.647043 | 0.517604 | 0.74 |
| Corynebacterium | 13803.23 | -0.27451 | 0.105833 | -2.5938 | 0.009492 | 0.08 |
| Polaromonas | 13341.73 | 0.04079 | 0.112948 | 0.361135 | 0.717998 | 0.86 |
| Vibrio | 13103.12 | 0.213136 | 0.100466 | 2.121483 | 0.033881 | 0.17 |
| Kitasatospora | 13004.91 | -0.2414 | 0.116395 | -2.07393 | 0.038086 | 0.18 |
| **Altererythrobacter** | **12743** | **0.28966** | **0.10324** | **2.80563** | **0.00502** | **0.05** |
| Cystobacter | 12684.17 | -0.01538 | 0.101832 | -0.15105 | 0.879936 | 0.95 |
| Roseimaritima | 12600.99 | 0.07221 | 0.104437 | 0.691419 | 0.489302 | 0.72 |
| Stenotrophomonas | 12577.87 | 0.206106 | 0.101766 | 2.025293 | 0.042837 | 0.19 |
| Aureimonas | 12461.31 | 0.200964 | 0.115329 | 1.742536 | 0.081415 | 0.27 |
| Acidobacterium | 12413.87 | 0.067327 | 0.115333 | 0.583765 | 0.559379 | 0.77 |
| Nitrosospira | 12351.38 | -0.11493 | 0.104914 | -1.09545 | 0.273318 | 0.53 |
| **Solimonas** | **12344** | **0.4116** | **0.12612** | **3.26383** | **0.0011** | **0.02** |
| **Gordonia** | **12243.4** | **-0.3635** | **0.11395** | **-3.1903** | **0.00142** | **0.02** |
| Edaphobacter | 12063.76 | -0.32125 | 0.13387 | -2.39987 | 0.016401 | 0.11 |
| Duganella | 12036.48 | 0.209042 | 0.104465 | 2.001075 | 0.045384 | 0.20 |
| Eimeria | 12032.99 | 0.01528 | 0.095016 | 0.160818 | 0.872237 | 0.95 |
| Paracoccus | 11915.24 | 0.117694 | 0.107511 | 1.094716 | 0.273641 | 0.53 |
| Rhizobacter | 11905.08 | -0.17888 | 0.108389 | -1.6503 | 0.098881 | 0.31 |
| Dyella | 11870.99 | 0.211833 | 0.111838 | 1.894119 | 0.058209 | 0.22 |
| Skermanella | 11856.88 | -0.07151 | 0.09617 | -0.74355 | 0.457148 | 0.70 |
| Oxytricha | 11852.08 | 0.234299 | 0.159875 | 1.470764 | 0.141355 | 0.37 |
| Haloferula | 11795.19 | -0.11206 | 0.092729 | -1.2085 | 0.226854 | 0.49 |
| Achromobacter | 11756.74 | 0.068339 | 0.105892 | 0.645363 | 0.518692 | 0.74 |
| Inquilinus | 11711.81 | 0.151598 | 0.106191 | 1.427594 | 0.153409 | 0.39 |
| Enhygromyxa | 11665.06 | 0.257355 | 0.138003 | 1.864951 | 0.062188 | 0.23 |
| Fimbriimonas | 11643.11 | 0.110031 | 0.132191 | 0.83237 | 0.4052 | 0.66 |
| Janthinobacterium | 11567.6 | 0.214275 | 0.098875 | 2.167138 | 0.030224 | 0.16 |
| Reyranella | 11564.87 | 0.114585 | 0.123889 | 0.924908 | 0.355014 | 0.62 |
| Herbaspirillum | 11393.7 | 0.189277 | 0.091713 | 2.063808 | 0.039036 | 0.18 |
| **Erythrobacter** | **11360** | **0.41602** | **0.10225** | **4.06883** | **4.72E-05** | **0.00** |
| Planctopirus | 11351.44 | 0.049629 | 0.107375 | 0.462198 | 0.643939 | 0.82 |
| Marinobacter | 11343.3 | 0.162728 | 0.096809 | 1.680925 | 0.092777 | 0.29 |
| Synechococcus | 11319.16 | -0.07 | 0.086635 | -0.808 | 0.419089 | 0.67 |
| Stylonychia | 11309.84 | 0.221444 | 0.15957 | 1.392355 | 0.163815 | 0.41 |
| Thioalkalivibrio | 11260.72 | 0.173718 | 0.104034 | 1.669822 | 0.094955 | 0.30 |
| Actinomyces | 11250.36 | -0.24214 | 0.110729 | -2.18675 | 0.028761 | 0.16 |
| **Streptacidiphilus** | **11138.9** | **-0.4065** | **0.12094** | **-3.361** | **0.00078** | **0.01** |
| Sinorhizobium | 11104.53 | -0.01192 | 0.099823 | -0.11945 | 0.904917 | 0.96 |
| Caballeronia | 10943.58 | -0.1582 | 0.105442 | -1.50035 | 0.133523 | 0.36 |
| **Nakamurella*** | **10885.8** | **-0.6717** | **0.16346** | **-4.1774** | **2.95E-05** | **0.00** |
| Emiliania | 10864.29 | -0.09694 | 0.10859 | -0.89271 | 0.37201 | 0.63 |
| Nevskia | 10818.43 | 0.148292 | 0.120978 | 1.225789 | 0.220278 | 0.48 |
| Cnuella | 10518.52 | 0.055452 | 0.155638 | 0.356293 | 0.721621 | 0.87 |
| Ralstonia | 10510.33 | -0.02567 | 0.090087 | -0.28499 | 0.77565 | 0.89 |
| Aspergillus | 10509.08 | -0.05786 | 0.099761 | -0.58 | 0.561917 | 0.77 |
| **Marmoricola** | **10501.1** | **-0.5349** | **0.15277** | **-3.5039** | **0.00046** | **0.01** |
| Ardenticatena | 10494.06 | 0.006499 | 0.097921 | 0.066367 | 0.947086 | 0.98 |
| Caldilinea | 10462.5 | 0.237499 | 0.11137 | 2.13254 | 0.032962 | 0.17 |
| Methylocapsa | 10013.55 | -0.08701 | 0.094733 | -0.91851 | 0.35835 | 0.63 |
| Arenimonas | 9769.042 | -0.04855 | 0.112217 | -0.43268 | 0.66525 | 0.83 |
| Rhizophagus | 9767.538 | 0.038109 | 0.117527 | 0.324769 | 0.745356 | 0.88 |
| **Patulibacter** | **9751.41** | **-0.5834** | **0.14635** | **-3.9883** | **6.65E-05** | **0.00** |
| **Roseiflexus** | **9723.57** | **-0.3047** | **0.08817** | **-3.4554** | **0.00055** | **0.01** |
| Methylocystis | 9684.171 | -0.06537 | 0.10112 | -0.64646 | 0.517983 | 0.74 |
| Anaerolinea | 9664.519 | 0.129212 | 0.131495 | 0.982645 | 0.325782 | 0.59 |
| Silvibacterium | 9557.9 | 0.035453 | 0.115279 | 0.307544 | 0.75843 | 0.88 |
| Sphingobacterium | 9397.729 | 0.105508 | 0.136194 | 0.774692 | 0.438522 | 0.69 |
| Filimonas | 9304.115 | 0.112748 | 0.151275 | 0.745344 | 0.456064 | 0.70 |
| Ramlibacter | 9301.909 | -0.22287 | 0.124136 | -1.79541 | 0.072588 | 0.25 |
| Leptospira | 9286.881 | 0.213116 | 0.108805 | 1.958716 | 0.050146 | 0.21 |
| Runella | 9219.959 | 0.171594 | 0.129352 | 1.326585 | 0.184646 | 0.43 |
| Plesiocystis | 9208.389 | 0.284497 | 0.131608 | 2.161784 | 0.030635 | 0.16 |
| **Phycicoccus** | **9126.72** | **-0.7745** | **0.14803** | **-5.2374** | **1.63E-07** | **0.00** |
| Belnapia | 9076.467 | -0.1821 | 0.112861 | -1.61345 | 0.106646 | 0.32 |
| Labrenzia | 9041.279 | 0.168263 | 0.111798 | 1.505074 | 0.132305 | 0.36 |
| Prevotella | 9011.351 | 0.100037 | 0.117003 | 0.855002 | 0.39255 | 0.65 |
| Acinetobacter | 8879.962 | 0.215581 | 0.102798 | 2.097143 | 0.035981 | 0.17 |
| Pseudogymnoascus | 8867.536 | 0.311796 | 0.113619 | 2.744252 | 0.006065 | 0.06 |
| Rubinisphaera | 8846.116 | 0.118394 | 0.106873 | 1.107801 | 0.267948 | 0.53 |
| Agrobacterium | 8829.737 | 0.200574 | 0.103532 | 1.937316 | 0.052707 | 0.21 |
| Lactobacillus | 8813.558 | -0.04409 | 0.089818 | -0.49091 | 0.623493 | 0.81 |
| Ensifer | 8800.044 | -0.07156 | 0.104461 | -0.68506 | 0.493304 | 0.72 |
| **Bdellovibrio** | **8700.57** | **0.47249** | **0.13226** | **3.57277** | **0.00035** | **0.01** |
| **Blastococcus** | **8683.15** | **-0.4542** | **0.13608** | **-3.3382** | **0.00084** | **0.01** |
| Terracidiphilus | 8675.414 | -0.07784 | 0.116292 | -0.66938 | 0.503254 | 0.73 |
| Candidatus Microthrix | 8656.879 | -0.30142 | 0.144973 | -2.07936 | 0.037584 | 0.18 |
| Chloroflexus | 8604.394 | -0.15358 | 0.089973 | -1.70693 | 0.087834 | 0.29 |
| Colletotrichum | 8599.061 | -0.18807 | 0.110179 | -1.70696 | 0.087829 | 0.29 |
| **Kribbella** | **8588.77** | **-0.701** | **0.14413** | **-4.8668** | **1.13E-06** | **0.00** |
| Pelomonas | 8585.206 | 0.294172 | 0.109488 | 2.686802 | 0.007214 | 0.06 |
| Thiobacillus | 8456.743 | 0.22775 | 0.099028 | 2.299849 | 0.021457 | 0.13 |
| Pseudoalteromonas | 8436.195 | 0.191813 | 0.098936 | 1.938767 | 0.05253 | 0.21 |
| Methylomonas | 8288.261 | 0.105213 | 0.10667 | 0.986347 | 0.323963 | 0.59 |
| Xanthobacter | 8247.589 | 0.167056 | 0.113304 | 1.47441 | 0.140371 | 0.37 |
| Pelagibacterium | 8226.325 | 0.225303 | 0.137447 | 1.639266 | 0.101158 | 0.31 |
| Alicyclobacillus | 8222.7 | -0.17139 | 0.091065 | -1.88204 | 0.05983 | 0.23 |
| **Hirschia**** | **8221.72** | **0.78251** | **0.14393** | **5.44071** | **5.31E-08** | **0.00** |
| Hydrogenophaga | 8168.875 | 0.054367 | 0.103426 | 0.525662 | 0.599123 | 0.80 |
| Rudaea | 8112.355 | 0.217022 | 0.141083 | 1.538338 | 0.123966 | 0.35 |
| Aureococcus | 8081.001 | -0.24016 | 0.12129 | -1.98006 | 0.047697 | 0.20 |
| Acetobacter | 8065.227 | 0.033458 | 0.097309 | 0.343834 | 0.730971 | 0.87 |
| Volvox | 8063.941 | -0.1268 | 0.102029 | -1.2428 | 0.213942 | 0.47 |
| Beijerinckia | 8036.517 | 0.108964 | 0.118789 | 0.917287 | 0.358992 | 0.63 |
| Pontibacter | 7994.57 | 0.085044 | 0.12389 | 0.686452 | 0.492428 | 0.72 |
| Desulfotomaculum | 7949.878 | -0.10789 | 0.09381 | -1.15005 | 0.250123 | 0.51 |
| Leifsonia | 7865.975 | 0.046961 | 0.12281 | 0.382388 | 0.702174 | 0.85 |
| **Rhodoferax** | **7844.5** | **0.5701** | **0.15099** | **3.77856** | **0.00016** | **0.00** |
| **Jiangella** | **7777.24** | **-0.5212** | **0.13476** | **-3.868** | **0.00011** | **0.00** |
| Thauera | 7716.581 | 0.094935 | 0.095438 | 0.994719 | 0.319873 | 0.59 |
| Rhodobacter | 7460.777 | 0.141289 | 0.093915 | 1.504443 | 0.132467 | 0.36 |
| **Acidimicrobium** | **7453.94** | **-0.4962** | **0.13531** | **-3.6673** | **0.00025** | **0.01** |
| Afifella | 7348.759 | 0.226865 | 0.120666 | 1.88014 | 0.060089 | 0.23 |
| Isosphaera | 7332.063 | -0.04107 | 0.092362 | -0.44463 | 0.656587 | 0.83 |
| Treponema | 7284.703 | 0.2548 | 0.108069 | 2.357774 | 0.018385 | 0.12 |
| Azoarcus | 7243.35 | 0.055337 | 0.102285 | 0.541008 | 0.588502 | 0.79 |
| **Saccharothrix** | **7219.29** | **-0.392** | **0.11205** | **-3.498** | **0.00047** | **0.01** |
| Rufibacter | 7184.18 | 0.112446 | 0.136235 | 0.825389 | 0.409151 | 0.66 |
| Hoeflea | 7172.697 | 0.296435 | 0.119668 | 2.477192 | 0.013242 | 0.10 |
| Flexibacter | 7156.976 | 0.064027 | 0.139711 | 0.458283 | 0.646749 | 0.82 |
| Azorhizobium | 7137.401 | 0.143682 | 0.117196 | 1.22601 | 0.220195 | 0.48 |
| Corallococcus | 7090.876 | 0.018164 | 0.097118 | 0.187033 | 0.851635 | 0.93 |
| Meiothermus | 7029.011 | -0.06659 | 0.094371 | -0.70558 | 0.480449 | 0.71 |
| Actinopolymorpha | 7018.882 | -0.34307 | 0.130787 | -2.62326 | 0.008709 | 0.07 |
| **Hydrocarboniphaga** | **7016.73** | **0.40316** | **0.13106** | **3.0764** | **0.0021** | **0.03** |
| Herpetosiphon | 6990.056 | -0.00089 | 0.107496 | -0.00832 | 0.993362 | 1.00 |
| Luteimonas | 6862.129 | 0.217151 | 0.11686 | 1.858236 | 0.063136 | 0.23 |
| Methylopila | 6849.972 | 0.190142 | 0.115347 | 1.648448 | 0.099261 | 0.31 |
| Pandoraea | 6813.176 | 0.141404 | 0.099754 | 1.417536 | 0.156326 | 0.39 |
| **Friedmanniella**** | **6802.88** | **-0.9758** | **0.15028** | **-6.5062** | **7.71E-11** | **0.00** |
| Oceanibaculum | 6789.477 | 0.198881 | 0.099862 | 1.99156 | 0.046419 | 0.20 |
| Chelatococcus | 6728.618 | 0.240208 | 0.107888 | 2.226468 | 0.025983 | 0.15 |
| Collimonas | 6727.831 | 0.243734 | 0.098419 | 2.476488 | 0.013268 | 0.10 |
| **Cephaloticoccus** | **6715.88** | **0.41844** | **0.14008** | **2.98757** | **0.00281** | **0.03** |
| Micromonas | 6674.145 | -0.1097 | 0.108901 | -1.00736 | 0.313762 | 0.58 |
| **Aeromicrobium** | **6663.24** | **-0.435** | **0.1404** | **-3.099** | **0.00194** | **0.03** |
| Plasmodium | 6637.404 | -0.17513 | 0.1263 | -1.38661 | 0.165561 | 0.41 |
| **Shewanella** | **6630.12** | **0.30868** | **0.10249** | **3.01177** | **0.0026** | **0.03** |
| Nitratireductor | 6570.549 | 0.176556 | 0.115655 | 1.526595 | 0.126862 | 0.35 |
| **Nitrososphaera** | **6550.57** | **-0.4088** | **0.1327** | **-3.3868** | **0.00071** | **0.01** |
| Thalassiosira | 6542.648 | -0.21205 | 0.108413 | -1.95591 | 0.050476 | 0.21 |
| Rubrivivax | 6497.348 | -0.14453 | 0.105271 | -1.37296 | 0.169765 | 0.41 |
| Spirochaeta | 6484.805 | 0.2204 | 0.120697 | 1.826082 | 0.067838 | 0.24 |
| Symbiodinium | 6483.927 | -0.05941 | 0.095212 | -0.62396 | 0.532652 | 0.75 |
| Prosthecomicrobium | 6474.771 | 0.246864 | 0.124565 | 1.98185 | 0.047496 | 0.20 |
| Thalassospira | 6468.878 | 0.268316 | 0.103921 | 2.58193 | 0.009825 | 0.08 |
| **Salinispora** | **6424.58** | **-0.3677** | **0.11169** | **-3.2918** | **0.001** | **0.02** |
| Phycisphaera | 6412.679 | 0.005625 | 0.112152 | 0.050154 | 0.96 | 0.98 |
| **Agromyces** | **6374.44** | **-0.3265** | **0.1181** | **-2.7645** | **0.0057** | **0.05** |
| **Woeseia** | **6372.61** | **0.54485** | **0.12119** | **4.49609** | **6.92E-06** | **0.00** |
| **Saccharopolyspora** | **6307.16** | **-0.3815** | **0.11989** | **-3.1817** | **0.00146** | **0.02** |
| Hydrotalea | 6298.502 | 0.045014 | 0.155863 | 0.28881 | 0.772726 | 0.89 |
| Nostoc | 6277.784 | -0.05947 | 0.106672 | -0.5575 | 0.577185 | 0.78 |
| Arachidicoccus | 6257.311 | 0.085944 | 0.150099 | 0.572594 | 0.566919 | 0.78 |
| Fulvivirga | 6249.625 | 0.07056 | 0.147787 | 0.47745 | 0.633042 | 0.82 |
| Haliscomenobacter | 6209.956 | 0.182771 | 0.144259 | 1.267033 | 0.205144 | 0.46 |
| **Tetrasphaera** | **6208.43** | **-0.6238** | **0.1391** | **-4.4856** | **7.27E-06** | **0.00** |
| Vulgatibacter | 6179.783 | 0.097449 | 0.111266 | 0.875825 | 0.381125 | 0.64 |
| Alcanivorax | 6008.775 | 0.243599 | 0.09966 | 2.444303 | 0.014513 | 0.10 |
| Desulfuromonas | 6007.809 | -0.17041 | 0.093175 | -1.82894 | 0.067409 | 0.24 |
| Curvibacter | 5986.213 | 0.042365 | 0.099005 | 0.427905 | 0.66872 | 0.83 |
| **Rubrobacter** | **5982.8** | **-0.5774** | **0.1238** | **-4.6639** | **3.10E-06** | **0.00** |
| Parapedobacter | 5929.786 | 0.083595 | 0.131796 | 0.634278 | 0.5259 | 0.75 |
| Labrys | 5929.754 | 0.125047 | 0.113496 | 1.101773 | 0.27056 | 0.53 |
| Acidiphilium | 5842.592 | -0.19383 | 0.108494 | -1.78654 | 0.074011 | 0.25 |
| Cryptosporangium | 5833.008 | -0.32233 | 0.130732 | -2.46566 | 0.013676 | 0.10 |
| Microbulbifer | 5805.339 | 0.099185 | 0.092485 | 1.072452 | 0.283517 | 0.55 |
| Pseudovibrio | 5787.428 | 0.17161 | 0.115337 | 1.487904 | 0.136776 | 0.37 |
| Sporichthya | 5718.946 | 0.055307 | 0.154762 | 0.357369 | 0.720815 | 0.87 |
| Roseovarius | 5707.373 | 0.146936 | 0.114347 | 1.285005 | 0.198791 | 0.45 |
| Rhodospirillum | 5696.842 | 0.203887 | 0.115701 | 1.762205 | 0.078035 | 0.26 |
| Blastochloris | 5658.162 | 0.174698 | 0.124525 | 1.402927 | 0.160639 | 0.40 |
| Rhodotorula | 5627.754 | -0.15952 | 0.105821 | -1.50742 | 0.131703 | 0.36 |
| Cyclobacterium | 5588.022 | 0.054112 | 0.109596 | 0.493747 | 0.621485 | 0.81 |
| **Dactylosporangium** | **5580.09** | **-0.5389** | **0.13631** | **-3.954** | **7.68E-05** | **0.00** |
| **Methylotenera**** | **5555.28** | **0.81104** | **0.13524** | **5.99905** | **1.98E-09** | **0.00** |
| Stappia | 5550.355 | 0.252022 | 0.120447 | 2.092413 | 0.036402 | 0.18 |
| Methylobacter | 5485.578 | -0.18935 | 0.126946 | -1.49156 | 0.135814 | 0.37 |
| Luteibacter | 5483.863 | 0.195858 | 0.116898 | 1.675479 | 0.09384 | 0.30 |
| Comamonas | 5446.264 | 0.065449 | 0.104903 | 0.623898 | 0.532695 | 0.75 |
| **Parvibaculum** | **5433.78** | **0.46016** | **0.11786** | **3.90443** | **9.44E-05** | **0.00** |
| Methyloversatilis | 5399.961 | 0.225114 | 0.102575 | 2.19464 | 0.028189 | 0.15 |
| **Cellvibrio** | **5396.27** | **0.49774** | **0.12023** | **4.14008** | **3.47E-05** | **0.00** |
| Marinobacterium | 5374.742 | 0.18931 | 0.100691 | 1.88011 | 0.060093 | 0.23 |
| Phyllobacterium | 5371.578 | 0.115155 | 0.119788 | 0.961334 | 0.336384 | 0.60 |
| Fusarium | 5322.462 | -0.15007 | 0.123708 | -1.21306 | 0.225106 | 0.48 |
| Thiomonas | 5305.341 | 0.107546 | 0.090916 | 1.182911 | 0.236845 | 0.50 |
| Saccharomonospora | 5301.374 | -0.28363 | 0.109489 | -2.59045 | 0.009585 | 0.08 |
| Chloracidobacterium | 5299.423 | -0.24833 | 0.125883 | -1.97268 | 0.048532 | 0.20 |
| **Microbispora** | **5280.01** | **-0.3696** | **0.11384** | **-3.2464** | **0.00117** | **0.02** |
| Lewinella | 5214.404 | 0.202114 | 0.107349 | 1.882792 | 0.059729 | 0.23 |
| Gloeobacter | 5200.191 | -0.07268 | 0.100689 | -0.72179 | 0.470426 | 0.71 |
| Thermus | 5200.033 | -0.25347 | 0.1046 | -2.42321 | 0.015384 | 0.10 |
| Lautropia | 5143.47 | 0.172938 | 0.103995 | 1.662947 | 0.096323 | 0.30 |
| Loktanella | 5136.956 | 0.23546 | 0.102902 | 2.288201 | 0.022126 | 0.13 |
| Curtobacterium | 5114.677 | -0.15823 | 0.130469 | -1.21276 | 0.225223 | 0.48 |
| Rubripirellula | 5100.085 | 0.193427 | 0.122226 | 1.582549 | 0.113524 | 0.33 |
| **Herbidospora** | **5078.68** | **-0.4085** | **0.13438** | **-3.0405** | **0.00236** | **0.03** |
| **Methyloligella** | **5077.47** | **-0.4551** | **0.12486** | **-3.6455** | **0.00027** | **0.01** |
| Azohydromonas | 5070.437 | -0.00699 | 0.107466 | -0.06505 | 0.948132 | 0.98 |
| Vitrella | 5065.583 | 0.048728 | 0.112331 | 0.433793 | 0.664439 | 0.83 |
| Pseudorhodoferax | 5056.605 | 0.051092 | 0.101541 | 0.503167 | 0.614847 | 0.81 |
| Gonium | 5049.166 | -0.05883 | 0.101451 | -0.5799 | 0.561984 | 0.77 |
| Bifidobacterium | 5019.2 | -0.2132 | 0.109604 | -1.94516 | 0.051755 | 0.21 |
| Aurantimonas | 5006.331 | 0.119667 | 0.107062 | 1.117742 | 0.263677 | 0.52 |
| Bryocella | 4988.099 | 0.237698 | 0.110292 | 2.155176 | 0.031148 | 0.16 |
| Penicillium | 4929.284 | -0.08844 | 0.104333 | -0.8477 | 0.396604 | 0.65 |
| **Thermoleophilum **** | **4895.58** | **-0.8565** | **0.15627** | **-5.496** | **3.89E-08** | **0.00** |
| Aeromonas | 4860.341 | 0.082995 | 0.104182 | 0.796634 | 0.425664 | 0.67 |
| Cyanothece | 4842.378 | -0.05826 | 0.111881 | -0.52075 | 0.602539 | 0.80 |
| Mizugakiibacter | 4798.57 | 0.277615 | 0.140323 | 1.978549 | 0.047867 | 0.20 |
| Ruegeria | 4794.17 | 0.014928 | 0.094218 | 0.158442 | 0.874109 | 0.95 |
| **Candidatus Nitrosocosmicus** | **4775.57** | **-0.3462** | **0.12654** | **-3.008** | **0.00263** | **0.03** |
| **Microtetraspora** | **4762.41** | **-0.4131** | **0.12431** | **-3.3236** | **0.00089** | **0.01** |
| Chthonomonas | 4737.491 | -0.14942 | 0.123964 | -1.20538 | 0.228057 | 0.49 |
| Sphaerobacter | 4734.125 | -0.27236 | 0.114184 | -2.38529 | 0.017066 | 0.11 |
| Trypanosoma | 4724.492 | 0.002847 | 0.100241 | 0.028403 | 0.977341 | 0.99 |
| Methylosinus | 4703.619 | -0.09396 | 0.102896 | -0.91313 | 0.361172 | 0.63 |
| Lysinimicrobium | 4701.976 | 0.22516 | 0.143286 | 1.571498 | 0.116067 | 0.34 |
| **Kutzneria** | **4679.14** | **-0.3283** | **0.11689** | **-2.8084** | **0.00498** | **0.05** |
| Thiothrix | 4665.692 | 0.276883 | 0.115624 | 2.394698 | 0.016634 | 0.11 |
| Pelobacter | 4656.851 | -0.23729 | 0.117095 | -2.02649 | 0.042714 | 0.19 |
| **Porphyrobacter** | **4656.49** | **0.29016** | **0.09533** | **3.0438** | **0.00234** | **0.03** |
| **Maricaulis** | **4655.25** | **0.44569** | **0.1308** | **3.40777** | **0.00065** | **0.01** |
| **Microlunatus** | **4639.69** | **-0.8171** | **0.13976** | **-5.8495** | **4.93E-09** | **0.00** |
| Tolypothrix | 4616.169 | 0.030487 | 0.104702 | 0.291174 | 0.770918 | 0.89 |
| Aquimarina | 4610.236 | 0.061626 | 0.131601 | 0.468282 | 0.639583 | 0.82 |
| Calothrix | 4595.833 | -0.0723 | 0.101377 | -0.71316 | 0.47575 | 0.71 |
| Pseudoxanthobacter | 4577.927 | 0.240411 | 0.128724 | 1.867699 | 0.061804 | 0.23 |
| Methanosarcina | 4575.547 | -0.27789 | 0.124162 | -2.23814 | 0.025212 | 0.14 |
| Ideonella | 4569.285 | -0.1819 | 0.114165 | -1.59328 | 0.111097 | 0.33 |
| Pleomorphomonas | 4556.928 | 0.215724 | 0.134347 | 1.605781 | 0.108322 | 0.32 |
| **Intrasporangium** | **4531.84** | **-0.7045** | **0.13966** | **-5.0463** | **4.50E-07** | **0.00** |
| Scytonema | 4525.804 | -0.15392 | 0.109305 | -1.40813 | 0.159091 | 0.40 |
| **Streptosporangium** | **4508.51** | **-0.5057** | **0.11811** | **-4.2817** | **1.85E-05** | **0.00** |
| Longilinea | 4507.457 | 0.129192 | 0.141764 | 0.91133 | 0.362122 | 0.63 |
| Demequina | 4469.713 | 0.277556 | 0.146461 | 1.895299 | 0.058053 | 0.22 |
| **Catenulispora** | **4456.42** | **-0.5336** | **0.13465** | **-3.9635** | **7.39E-05** | **0.00** |
| Brevibacillus | 4439.843 | -0.02853 | 0.100185 | -0.28477 | 0.775823 | 0.89 |
| Catalinimonas | 4428.718 | 0.111235 | 0.126669 | 0.878168 | 0.379852 | 0.64 |
| Filomicrobium | 4398.003 | 0.060523 | 0.112334 | 0.538774 | 0.590043 | 0.79 |
| Sulfitobacter | 4363.323 | 0.126026 | 0.112365 | 1.121575 | 0.262043 | 0.52 |
| Geminicoccus | 4293.665 | 0.079094 | 0.115875 | 0.682579 | 0.494873 | 0.72 |
| Thermogemmatispora | 4282.149 | -0.23244 | 0.105673 | -2.19956 | 0.027838 | 0.15 |
| Ectocarpus | 4274.235 | -0.0305 | 0.09798 | -0.31124 | 0.755618 | 0.88 |
| **Modestobacter** | **4261.82** | **-0.4382** | **0.12408** | **-3.5314** | **0.00041** | **0.01** |
| Oscillatoria | 4252.303 | -0.00655 | 0.106234 | -0.06169 | 0.950811 | 0.98 |
| Noviherbaspirillum | 4241.939 | 0.184492 | 0.108709 | 1.697114 | 0.089675 | 0.29 |
| Dictyostelium | 4238.564 | 0.075593 | 0.128589 | 0.587869 | 0.55662 | 0.77 |
| Terrimicrobium | 4209.816 | 0.006055 | 0.12169 | 0.04976 | 0.960314 | 0.98 |
| Thiocapsa | 4180.913 | -0.12992 | 0.10801 | -1.20287 | 0.229028 | 0.49 |
| Frateuria | 4175.499 | 0.273687 | 0.133374 | 2.052102 | 0.04016 | 0.18 |
| **Janibacter** | **4170.15** | **-0.4787** | **0.13356** | **-3.5842** | **0.00034** | **0.01** |
| Desulfobulbus | 4163.645 | 0.047766 | 0.103753 | 0.460383 | 0.645241 | 0.82 |
| Lentzea | 4158.669 | -0.26434 | 0.115085 | -2.29694 | 0.021622 | 0.13 |
| Rhodomicrobium | 4152.203 | -0.25575 | 0.106997 | -2.39029 | 0.016835 | 0.11 |
| Sporocytophaga | 4141.013 | 0.014875 | 0.132036 | 0.11266 | 0.9103 | 0.96 |
| Elioraea | 4116.018 | 0.035636 | 0.104674 | 0.340447 | 0.73352 | 0.87 |
| Streptococcus | 4105.771 | -0.01482 | 0.10028 | -0.14783 | 0.882476 | 0.95 |
| Aminobacter | 4099.754 | 0.306039 | 0.113904 | 2.686841 | 0.007213 | 0.06 |
| Acidisphaera | 4099.421 | -0.04878 | 0.113669 | -0.42915 | 0.667816 | 0.83 |
| Chlorobium | 4059.684 | 0.052723 | 0.09697 | 0.543702 | 0.586647 | 0.79 |
| Rhizoctonia | 4044.447 | -0.14403 | 0.111903 | -1.28711 | 0.198057 | 0.45 |
| Nitrosococcus | 4027.061 | 0.110531 | 0.101935 | 1.084329 | 0.278219 | 0.54 |
| Photobacterium | 4015.877 | 0.070825 | 0.096976 | 0.730336 | 0.465185 | 0.71 |
| **Oceanicaulis** | **4005.24** | **0.48205** | **0.12787** | **3.77014** | **0.00016** | **0.00** |
| Chlamydomonas | 3995.923 | -0.02217 | 0.114922 | -0.19289 | 0.847047 | 0.93 |
| Phaeospirillum | 3993.111 | 0.072546 | 0.11429 | 0.634758 | 0.525587 | 0.75 |
| Monoraphidium | 3991.065 | -0.08032 | 0.103782 | -0.77393 | 0.438972 | 0.69 |
| Alistipes | 3987.952 | 0.091867 | 0.104152 | 0.882043 | 0.377754 | 0.64 |
| Lutibaculum | 3960.461 | 0.196473 | 0.131325 | 1.49611 | 0.134625 | 0.37 |
| Dechloromonas | 3933.449 | 0.217124 | 0.163215 | 1.332631 | 0.182653 | 0.43 |
| Methyloferula | 3931.035 | 0.102673 | 0.11893 | 0.863301 | 0.387972 | 0.65 |
| **Kordiimonas** | **3913.65** | **0.30989** | **0.10831** | **2.86101** | **0.00422** | **0.05** |
| Monosiga | 3908.762 | 0.036603 | 0.148648 | 0.246242 | 0.805495 | 0.91 |
| Allomyces | 3882.978 | -0.0594 | 0.115735 | -0.51326 | 0.607773 | 0.80 |
| Fluviicola | 3867.081 | 0.075474 | 0.149465 | 0.504974 | 0.613577 | 0.81 |
| Levilinea | 3856.177 | 0.019078 | 0.134963 | 0.14136 | 0.887586 | 0.95 |
| Jannaschia | 3851.742 | 0.119562 | 0.113459 | 1.053801 | 0.291974 | 0.55 |
| Escherichia | 3847.635 | -0.00402 | 0.106688 | -0.03768 | 0.969944 | 0.99 |
| Sediminibacterium | 3845.057 | 0.156823 | 0.15821 | 0.991362 | 0.321509 | 0.59 |
| **Ponticaulis** | **3833.24** | **0.72256** | **0.12976** | **5.56944** | **2.56E-08** | **0.00** |
| Ancylobacter | 3832.332 | 0.10526 | 0.119554 | 0.880451 | 0.378615 | 0.64 |
| **Chelativorans** | **3824.76** | **0.37562** | **0.11737** | **3.20031** | **0.00137** | **0.02** |
| **Hamadaea** | **3822.5** | **-0.5187** | **0.13147** | **-3.9457** | **7.96E-05** | **0.00** |
| **Terrabacter **** | **3812.63** | **-0.9076** | **0.15181** | **-5.9899** | **2.10E-09** | **0.00** |
| **Actinokineospora** | **3798.72** | **-0.36** | **0.13016** | **-2.7658** | **0.00568** | **0.05** |
| Ruminococcus | 3778.97 | -0.02306 | 0.102464 | -0.22502 | 0.821965 | 0.92 |
| Auxenochlorella | 3750.929 | -0.23231 | 0.116296 | -1.9976 | 0.04576 | 0.20 |
| Tistrella | 3746.021 | 0.226659 | 0.10122 | 2.239277 | 0.025138 | 0.14 |
| Exophiala | 3724.498 | -0.13469 | 0.104536 | -1.28847 | 0.197584 | 0.45 |
| Chromobacterium | 3713.799 | 0.081153 | 0.10434 | 0.777773 | 0.436703 | 0.69 |
| Desulfosporosinus | 3694.556 | -0.14558 | 0.107776 | -1.35077 | 0.17677 | 0.42 |
| Flexithrix | 3680.861 | 0.11996 | 0.125504 | 0.955841 | 0.339153 | 0.61 |
| Oscillochloris | 3674.128 | -0.15091 | 0.109299 | -1.38074 | 0.16736 | 0.41 |
| Sulfurifustis | 3671.489 | 0.070259 | 0.105035 | 0.668913 | 0.503551 | 0.73 |
| Serratia | 3668.985 | 0.127523 | 0.106598 | 1.196292 | 0.231583 | 0.49 |
| Asinibacterium | 3667.59 | 0.102896 | 0.155548 | 0.661534 | 0.50827 | 0.74 |
| Kocuria | 3652.901 | -0.26492 | 0.119163 | -2.22316 | 0.026205 | 0.15 |
| Thiohalocapsa | 3650.506 | -0.04076 | 0.120956 | -0.33701 | 0.736107 | 0.87 |
| Croceicoccus | 3641.518 | 0.332828 | 0.121342 | 2.742949 | 0.006089 | 0.06 |
| Kiloniella | 3632.19 | 0.234188 | 0.111363 | 2.102947 | 0.03547 | 0.17 |
| Albimonas | 3616.88 | 0.13274 | 0.132053 | 1.005222 | 0.31479 | 0.58 |
| Ochrobactrum | 3610.569 | 0.128235 | 0.113503 | 1.129796 | 0.258562 | 0.52 |
| Beggiatoa | 3591.217 | 0.187808 | 0.115683 | 1.62347 | 0.104489 | 0.32 |
| Tardiphaga | 3587.65 | 0.16578 | 0.119182 | 1.390982 | 0.164231 | 0.41 |
| Acanthamoeba | 3584.395 | 0.036935 | 0.103833 | 0.35572 | 0.722051 | 0.87 |
| **Tepidicaulis** | **3584.27** | **0.35544** | **0.12386** | **2.86988** | **0.00411** | **0.04** |
| Rheinheimera | 3572.287 | 0.285129 | 0.11708 | 2.435348 | 0.014877 | 0.10 |
| Methylobrevis | 3568.641 | 0.331268 | 0.131774 | 2.514046 | 0.011935 | 0.09 |
| Geothrix | 3567.326 | -0.20332 | 0.12801 | -1.58833 | 0.112212 | 0.33 |
| **Nitriliruptor** | **3556.18** | **-0.4423** | **0.13213** | **-3.3472** | **0.00082** | **0.01** |
| Nitrolancea | 3548.895 | -0.21663 | 0.124567 | -1.73902 | 0.082031 | 0.27 |
| Salpingoeca | 3533.563 | -0.14736 | 0.108695 | -1.35569 | 0.175197 | 0.42 |
| Rhodothermus | 3521.355 | -0.22834 | 0.095196 | -2.39861 | 0.016457 | 0.11 |
| Kibdelosporangium | 3513.356 | -0.26702 | 0.112087 | -2.38226 | 0.017207 | 0.11 |
| **Alteromonas** | **3511.88** | **0.35566** | **0.11522** | **3.08692** | **0.00202** | **0.03** |
| Pajaroellobacter | 3507.145 | 0.290513 | 0.132468 | 2.193181 | 0.028294 | 0.15 |
| **Asanoa** | **3504.88** | **-0.5402** | **0.13548** | **-3.9878** | **6.67E-05** | **0.00** |
| Maribacter | 3504.85 | 0.039953 | 0.131672 | 0.303431 | 0.761561 | 0.88 |
| **Methylobacillus** | **3501.81** | **0.49587** | **0.09614** | **5.15784** | **2.50E-07** | **0.00** |
| Bartonella | 3501.518 | 0.290368 | 0.127091 | 2.284813 | 0.022324 | 0.13 |
| Fabibacter | 3497.821 | 0.121686 | 0.139476 | 0.87247 | 0.382952 | 0.64 |
| Pseudohongiella | 3495.128 | 0.254639 | 0.120374 | 2.11541 | 0.034395 | 0.17 |
| Ornatilinea | 3489.992 | 0.091941 | 0.139933 | 0.657036 | 0.511158 | 0.74 |
| Candidatus Brocadia | 3483.099 | -0.31368 | 0.1215 | -2.58175 | 0.00983 | 0.08 |
| **Actinophytocola** | **3479.75** | **-0.4351** | **0.12031** | **-3.6165** | **0.0003** | **0.01** |
| Methylocella | 3475.277 | 0.002857 | 0.121597 | 0.023496 | 0.981255 | 0.99 |
| Glycomyces | 3466.018 | -0.29319 | 0.11956 | -2.45224 | 0.014197 | 0.10 |
| Mitsuaria | 3451.419 | -0.00354 | 0.111713 | -0.03172 | 0.974699 | 0.99 |
| Sporothrix | 3446.711 | -0.16861 | 0.130868 | -1.28839 | 0.19761 | 0.45 |
| Ruminiclostridium | 3445.317 | -0.10277 | 0.1054 | -0.97506 | 0.329528 | 0.60 |
| Roseivirga | 3439.553 | 0.098726 | 0.130758 | 0.755043 | 0.450223 | 0.70 |
| Puccinia | 3430.482 | -0.09317 | 0.111268 | -0.83738 | 0.402377 | 0.66 |
| Caenimonas | 3425.983 | -0.19087 | 0.164112 | -1.16424 | 0.244325 | 0.51 |
| Starkeya | 3424.858 | 0.120075 | 0.119225 | 1.007125 | 0.313875 | 0.58 |
| Oligotropha | 3405.043 | 0.283539 | 0.129639 | 2.187187 | 0.028729 | 0.16 |
| Chitinimonas | 3377.225 | 0.228096 | 0.105763 | 2.156685 | 0.03103 | 0.16 |
| Microcystis | 3365.215 | -0.1117 | 0.118476 | -0.9428 | 0.345785 | 0.61 |
| Marinomonas | 3355.847 | 0.190821 | 0.101816 | 1.874177 | 0.060906 | 0.23 |
| Propionivibrio | 3350.452 | 0.022625 | 0.105487 | 0.214483 | 0.83017 | 0.92 |
| **Lechevalieria** | **3328.25** | **-0.3667** | **0.12918** | **-2.8387** | **0.00453** | **0.05** |
| Cytophaga | 3322.168 | 0.097265 | 0.145354 | 0.66918 | 0.503381 | 0.73 |
| Martelella | 3317.999 | 0.223766 | 0.122842 | 1.82161 | 0.068514 | 0.24 |
| Cryobacterium | 3315.345 | -0.07098 | 0.139401 | -0.50917 | 0.61063 | 0.81 |
| Thalassobaculum | 3283.908 | 0.18941 | 0.117379 | 1.613677 | 0.106598 | 0.32 |
| Thermobaculum | 3277.999 | -0.1996 | 0.112705 | -1.77095 | 0.076569 | 0.26 |
| Enterococcus | 3271.997 | -0.032 | 0.103192 | -0.31009 | 0.756493 | 0.88 |
| Candidatus Contendobacter | 3254.775 | 0.078529 | 0.118361 | 0.663469 | 0.50703 | 0.73 |
| **Alloactinosynnema** | **3247.93** | **-0.3749** | **0.13282** | **-2.8228** | **0.00476** | **0.05** |
| **Knoellia** | **3240.79** | **-0.584** | **0.13864** | **-4.2136** | **2.51E-05** | **0.00** |
| Acidithiobacillus | 3219.56 | 0.025446 | 0.113642 | 0.223913 | 0.822825 | 0.92 |
| Acidocella | 3194.944 | 0.195089 | 0.109162 | 1.787163 | 0.073911 | 0.25 |
| Mortierella | 3187.129 | -0.25725 | 0.111658 | -2.30393 | 0.021227 | 0.13 |
| Leptolinea | 3172.12 | 0.083024 | 0.138222 | 0.600655 | 0.54807 | 0.76 |
| Fibrisoma | 3169.94 | -0.00184 | 0.142498 | -0.01289 | 0.989715 | 0.99 |
| **Candidatus Methylomirabilis** | **3149.36** | **-0.3943** | **0.12274** | **-3.2124** | **0.00132** | **0.02** |
| Endozoicomonas | 3129.048 | 0.183821 | 0.112347 | 1.63619 | 0.1018 | 0.31 |
| Pseudanabaena | 3114.1 | -0.0741 | 0.117477 | -0.6308 | 0.528173 | 0.75 |
| Pantoea | 3109.745 | 0.16309 | 0.112066 | 1.455315 | 0.145582 | 0.38 |
| Phormidium | 3105.746 | -0.01199 | 0.112772 | -0.10634 | 0.915313 | 0.97 |
| Salinarimonas | 3104.273 | 0.053738 | 0.113796 | 0.472224 | 0.636767 | 0.82 |
| Leishmania | 3103.023 | 0.010788 | 0.103083 | 0.104652 | 0.916652 | 0.97 |
| Rubritalea | 3089.149 | -0.10551 | 0.115551 | -0.91315 | 0.361165 | 0.63 |
| Pararhodospirillum | 3088.667 | 0.294777 | 0.109676 | 2.687701 | 0.007195 | 0.06 |
| Acidihalobacter | 3082.014 | 0.29447 | 0.117668 | 2.502574 | 0.012329 | 0.09 |
| Fontimonas | 3077.43 | 0.188049 | 0.144092 | 1.305111 | 0.191855 | 0.44 |
| Nitrosovibrio | 3072.65 | -0.05304 | 0.094973 | -0.55851 | 0.576493 | 0.78 |
| Leptospirillum | 3059.039 | -0.02168 | 0.096126 | -0.22554 | 0.821555 | 0.92 |
| Rubellimicrobium | 3055.11 | 0.145905 | 0.110911 | 1.315525 | 0.188333 | 0.43 |
| Siphonobacter | 3051.158 | 0.217481 | 0.125929 | 1.727053 | 0.084158 | 0.28 |
| Emticicia | 3042.823 | 0.1325 | 0.14568 | 0.909564 | 0.363052 | 0.63 |
| Eubacterium | 3035.216 | 0.014848 | 0.099507 | 0.14921 | 0.881388 | 0.95 |
| **Candidatus Phaeomarinobacter** | **3031.65** | **0.37813** | **0.11821** | **3.19881** | **0.00138** | **0.02** |
| Arcticibacter | 3030.024 | 0.071709 | 0.145796 | 0.491859 | 0.622819 | 0.81 |
| Phytophthora | 3028.89 | 0.123604 | 0.110909 | 1.114464 | 0.26508 | 0.52 |
| **Turneriella** | **3024.18** | **0.59006** | **0.15566** | **3.7951** | **0.00015** | **0.00** |
| Solitalea | 3021.231 | 0.06671 | 0.141095 | 0.472807 | 0.636351 | 0.82 |
| Amorphus | 3017.311 | 0.331122 | 0.134823 | 2.456115 | 0.014045 | 0.10 |
| **Idiomarina** | **3001.46** | **0.41192** | **0.1054** | **3.90817** | **9.30E-05** | **0.00** |
| Salinisphaera | 2981.237 | 0.020089 | 0.12964 | 0.154962 | 0.876851 | 0.95 |
| Pseudarcicella | 2971.692 | 0.100759 | 0.151634 | 0.664517 | 0.506359 | 0.73 |
| Thecamonas | 2960.441 | -0.00933 | 0.116534 | -0.08004 | 0.936201 | 0.98 |
| Methylosarcina | 2958.928 | -0.0907 | 0.123036 | -0.7372 | 0.460998 | 0.70 |
| Adhaeribacter | 2957.953 | 0.189165 | 0.142008 | 1.332143 | 0.182813 | 0.43 |
| Fodinicurvata | 2951.253 | 0.184435 | 0.114672 | 1.608381 | 0.107752 | 0.32 |
| Trichoderma | 2941.089 | -0.1879 | 0.135533 | -1.38643 | 0.165617 | 0.41 |
| Polymorphum | 2931.902 | 0.250655 | 0.129808 | 1.931027 | 0.05348 | 0.22 |
| Arsenicibacter | 2908.77 | 0.124584 | 0.142325 | 0.875367 | 0.381374 | 0.64 |
| Limnohabitans | 2905.043 | 0.047522 | 0.117736 | 0.403638 | 0.686479 | 0.84 |
| Teredinibacter | 2904.536 | 0.260805 | 0.11572 | 2.253792 | 0.024209 | 0.14 |
| Bellilinea | 2899.959 | -0.10085 | 0.126057 | -0.80003 | 0.423693 | 0.67 |
| Verticillium | 2899.54 | -0.1721 | 0.126928 | -1.35588 | 0.175139 | 0.42 |

Table S4: Functional categories annotation of reads

|  | **Metabolism** | **Genetic Information Processing** | **Environmental Information Processing** | **Cellular Processes** | **Organismal Systems** | **Human Diseases** | **Unclassified** | **Not assigned** |
| --- | --- | --- | --- | --- | --- | --- | --- | --- |
| ARD_1_Bulk | 331165 | 84389 | 76371 | 26774 | 14550 | 19570 | 350512 | 526243 |
| ARD_2_Bulk | 513991 | 119971 | 116265 | 39668 | 22037 | 28114 | 507113 | 724834 |
| ARD_1_Bulk | 455729 | 110522 | 104972 | 36654 | 19892 | 25627 | 459146 | 655876 |
| ARD_2_Bulk | 510025 | 117737 | 114327 | 39587 | 21138 | 26666 | 498462 | 690573 |
| CO_1_Bulk | 368723 | 85489 | 81863 | 27060 | 15651 | 19336 | 359551 | 512752 |
| CO_2_Bulk | 459037 | 112700 | 105744 | 36629 | 20238 | 25926 | 476465 | 712552 |
| CO_1_Bulk | 406140 | 93233 | 90081 | 30063 | 16654 | 20937 | 392835 | 557615 |
| CO_2_Bulk | 461784 | 108928 | 107436 | 36464 | 19711 | 25279 | 459725 | 652603 |
| ARD_1_Rhizo | 421896 | 104452 | 103844 | 38141 | 18362 | 24484 | 423886 | 566258 |
| ARD_2_Rhizo | 397577 | 100560 | 95618 | 35669 | 17912 | 23851 | 406392 | 564303 |
| ARD_1_Rhizo | 431944 | 103055 | 102943 | 37636 | 18730 | 23991 | 435946 | 591797 |
| ARD_2_Rhizo | 448206 | 108133 | 111744 | 40992 | 19792 | 25503 | 447646 | 584984 |
| CO_1_Rhizo | 437707 | 102830 | 101542 | 35477 | 18800 | 23880 | 428143 | 595864 |
| CO_2_Rhizo | 526939 | 123705 | 122917 | 42375 | 22220 | 28138 | 517976 | 720597 |
| CO_3_Rhizo | 460642 | 111848 | 109174 | 39793 | 19974 | 26022 | 465281 | 652839 |
| CO_3_Rhizo | 554790 | 131383 | 130979 | 44550 | 24120 | 30457 | 552315 | 789096 |

Table S5: Results from DESeq2 analysis of rhizosphere samples based on functional annotation of the overall data. Table shows pairwise comparisons between the treatments using control as base (only reads with p < 0.05) are shown.

| **DESeq2 Functional annotation** | | | |  |  |  |
| --- | --- | --- | --- | --- | --- | --- |
| ***KO*** | ***baseMean*** | ***log2FoldChange*** | ***lfcSE*** | ***stat*** | ***pvalue*** | ***padj*** |
| K01844 | 40.63974 | -0.18348 | 0.050958 | -3.62809 | 0.000286 | 0.64872 |
| K01601 | 77.12926 | -0.19852 | 0.062371 | -3.18168 | 0.001464 | 0.999684 |
| K01061 | 889.6305 | 0.157966 | 0.052941 | 2.983446 | 0.00285 | 0.999684 |
| K03921 | 83.30796 | -0.18118 | 0.060836 | -2.97401 | 0.002939 | 0.999684 |
| K02416 | 127.474 | 0.188504 | 0.065153 | 2.889563 | 0.003858 | 0.999684 |
| K01779 | 244.7397 | 0.182732 | 0.06578 | 2.779309 | 0.005447 | 0.999684 |
| K03883 | 12.64602 | 0.085702 | 0.032209 | 2.736571 | 0.006208 | 0.999684 |
| K10232 | 84.85256 | -0.16319 | 0.061296 | -2.66374 | 0.007728 | 0.999684 |
| K01114 | 471.9096 | -0.15371 | 0.060209 | -2.55266 | 0.01069 | 0.999684 |
| K16148 | 122.332 | -0.16522 | 0.065412 | -2.52499 | 0.01157 | 0.999684 |
| K01081 | 295.0289 | -0.16126 | 0.063977 | -2.52045 | 0.01172 | 0.999684 |
| K12541 | 58.04606 | 0.149385 | 0.059611 | 2.505192 | 0.012239 | 0.999684 |
| K00472 | 21.86558 | 0.108547 | 0.044475 | 2.450418 | 0.014269 | 0.999684 |
| K02474 | 191.0283 | 0.151936 | 0.062936 | 2.418636 | 0.015579 | 0.999684 |
| K07716 | 371.3815 | 0.154981 | 0.064292 | 2.409246 | 0.015986 | 0.999684 |
| K10804 | 122.6792 | 0.153033 | 0.064795 | 2.363447 | 0.018106 | 0.999684 |
| K01760 | 244.9643 | 0.153496 | 0.065388 | 2.346601 | 0.018946 | 0.999684 |
| K10800 | 24.19625 | -0.09395 | 0.040446 | -2.33977 | 0.019295 | 0.999684 |
| K14257 | 62.59783 | 0.13188 | 0.056712 | 2.332576 | 0.01967 | 0.999684 |
| K00114 | 1532.861 | 0.144457 | 0.062389 | 2.315678 | 0.020576 | 0.999684 |
| K05588 | 26.94803 | 0.099714 | 0.043703 | 2.307135 | 0.021047 | 0.999684 |
| K01207 | 398.4749 | 0.146528 | 0.064133 | 2.284625 | 0.022335 | 0.999684 |
| K13039 | 43.85169 | 0.121587 | 0.05423 | 2.248009 | 0.024576 | 0.999684 |
| K00856 | 218.5379 | -0.14719 | 0.065761 | -2.23705 | 0.025283 | 0.999684 |
| K00141 | 54.06016 | -0.12147 | 0.05451 | -2.22762 | 0.025906 | 0.999684 |
| K02965 | 205.7222 | 0.145837 | 0.065482 | 2.225972 | 0.026016 | 0.999684 |
| K13542 | 247.183 | -0.1448 | 0.065485 | -2.21294 | 0.026902 | 0.999684 |
| K04098 | 52.86274 | -0.12609 | 0.057192 | -2.20189 | 0.027673 | 0.999684 |
| K02878 | 242.5439 | -0.14322 | 0.065218 | -2.19605 | 0.028089 | 0.999684 |
| K05522 | 91.99851 | -0.13834 | 0.063224 | -2.18812 | 0.028661 | 0.999684 |
| K01505 | 56.84484 | -0.12396 | 0.057017 | -2.17344 | 0.029747 | 0.999684 |
| K06861 | 314.239 | 0.142024 | 0.065512 | 2.167961 | 0.030162 | 0.999684 |
| K02408 | 34.83203 | 0.105277 | 0.049193 | 2.143159 | 0.0321 | 0.999684 |
| K01950 | 639.7734 | -0.12298 | 0.057403 | -2.14236 | 0.032165 | 0.999684 |
| K09882 | 106.3412 | 0.13741 | 0.064827 | 2.118631 | 0.034122 | 0.999684 |
| K01222 | 100.5989 | -0.11145 | 0.052857 | -2.11358 | 0.034551 | 0.999684 |
| K01431 | 20.79273 | 0.094978 | 0.045169 | 2.109067 | 0.034939 | 0.999684 |
| K07660 | 23.51427 | 0.099604 | 0.047486 | 2.100065 | 0.035723 | 0.999684 |
| K03196 | 57.02166 | 0.116299 | 0.05553 | 2.093444 | 0.03631 | 0.999684 |
| K16012 | 32.88736 | -0.09444 | 0.045526 | -2.08863 | 0.036741 | 0.999684 |
| K02902 | 144.7898 | 0.136021 | 0.065181 | 2.087142 | 0.036875 | 0.999684 |
| K14519 | 136.4388 | -0.13305 | 0.063995 | -2.08072 | 0.03746 | 0.999684 |
| K11003 | 73.1533 | 0.12857 | 0.062053 | 2.07027 | 0.038427 | 0.999684 |
| K01889 | 596.4533 | 0.12041 | 0.05828 | 2.0659 | 0.038838 | 0.999684 |
| K07806 | 82.3964 | -0.1293 | 0.062864 | -2.05937 | 0.039459 | 0.999684 |
| K08683 | 13.32467 | -0.06812 | 0.033557 | -2.05412 | 0.039964 | 0.999684 |
| K10552 | 35.70032 | -0.09136 | 0.044743 | -2.05188 | 0.040181 | 0.999684 |
| K09694 | 102.3931 | -0.12621 | 0.061705 | -2.04623 | 0.040733 | 0.999684 |
| K02388 | 70.20074 | 0.11415 | 0.055978 | 2.039545 | 0.041396 | 0.999684 |
| K02651 | 54.88804 | 0.110482 | 0.055659 | 1.991276 | 0.046451 | 0.999684 |
| K02551 | 68.32685 | -0.10935 | 0.055358 | -1.9731 | 0.048484 | 0.999684 |


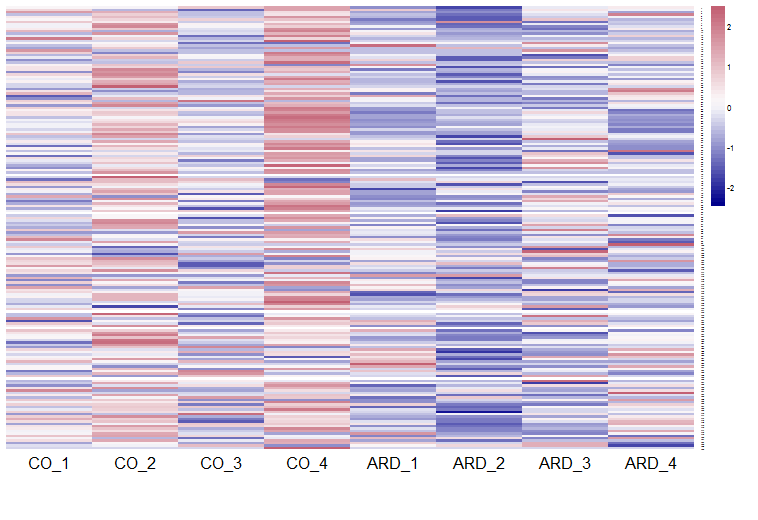


Figure S1: Non-negative matrix factorization analysis of functional annotation (KEGG levels 5) of reads assigned to category xenobiotic metabolism.


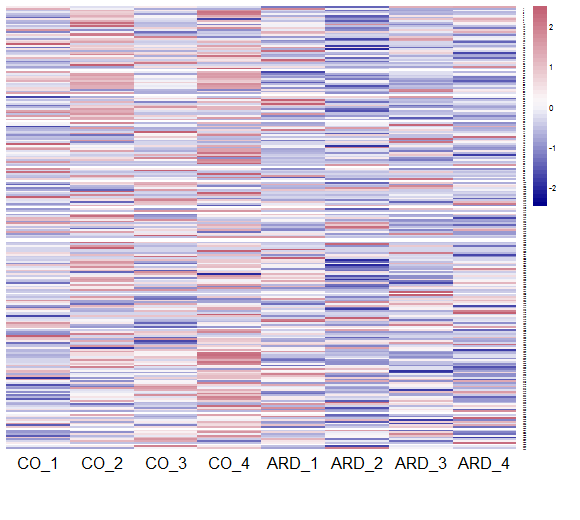


Figure S2: Non-negative matrix factorization analysis of functional annotation (KEGG levels 5) of reads assigned to the category metabolism of terpenoids and polyketides.

Figure S3 a


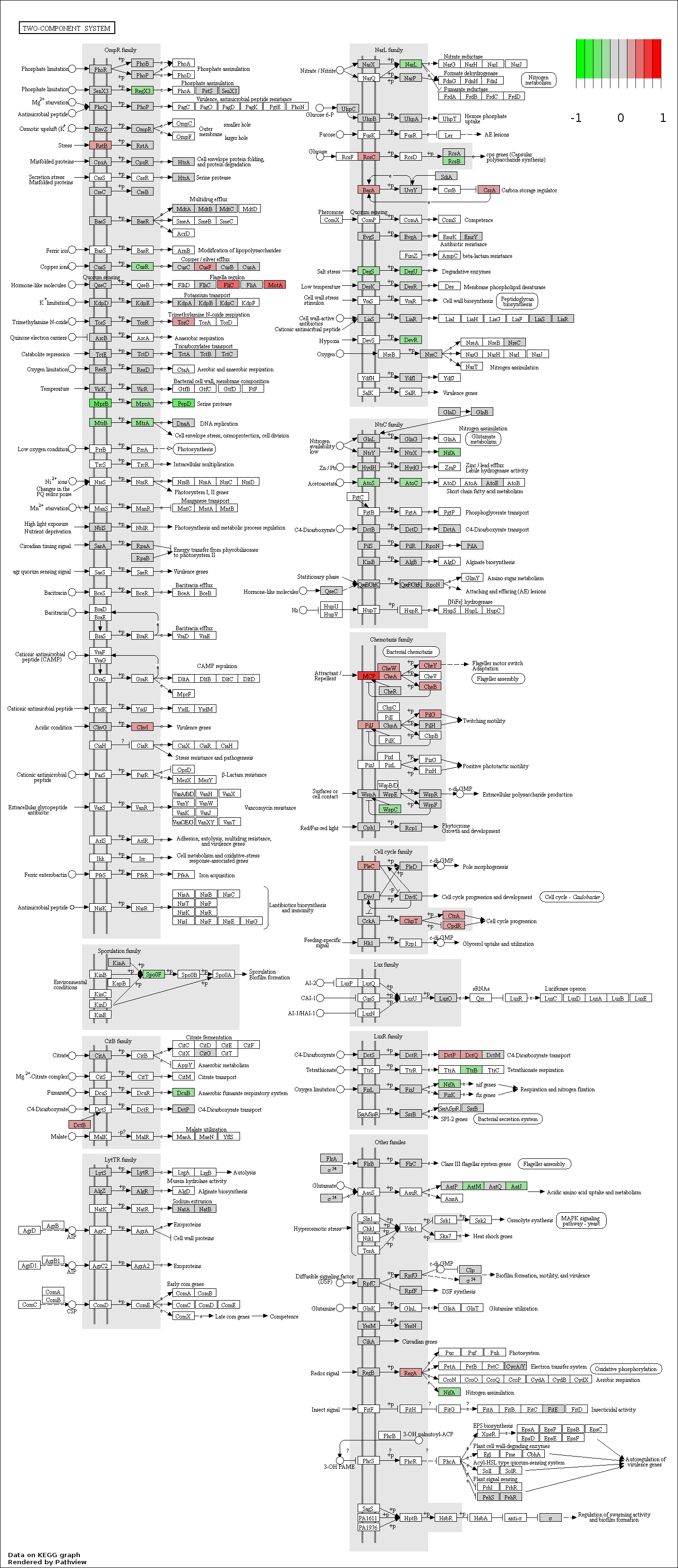


Figure S3 b


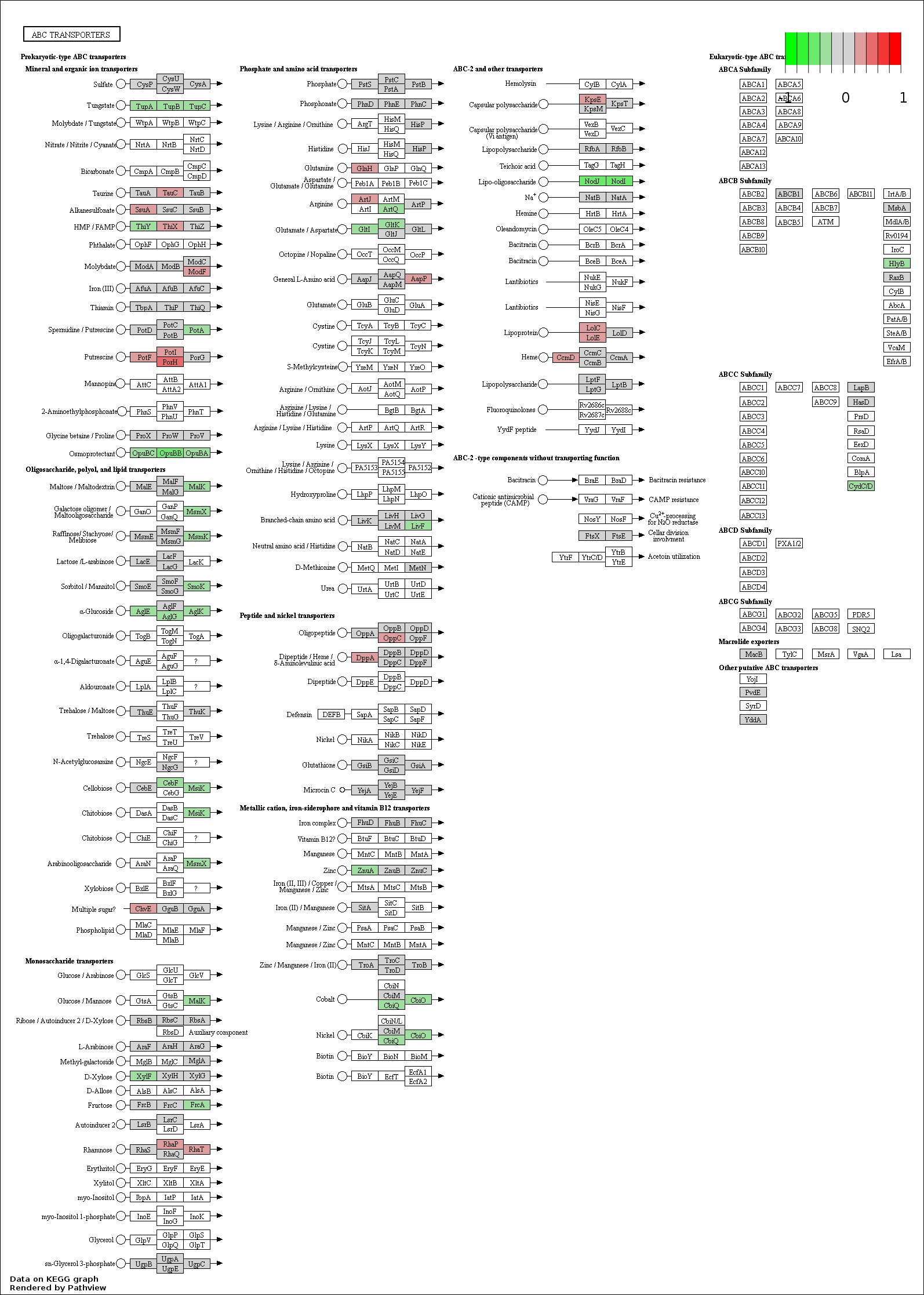


Figure S3 c


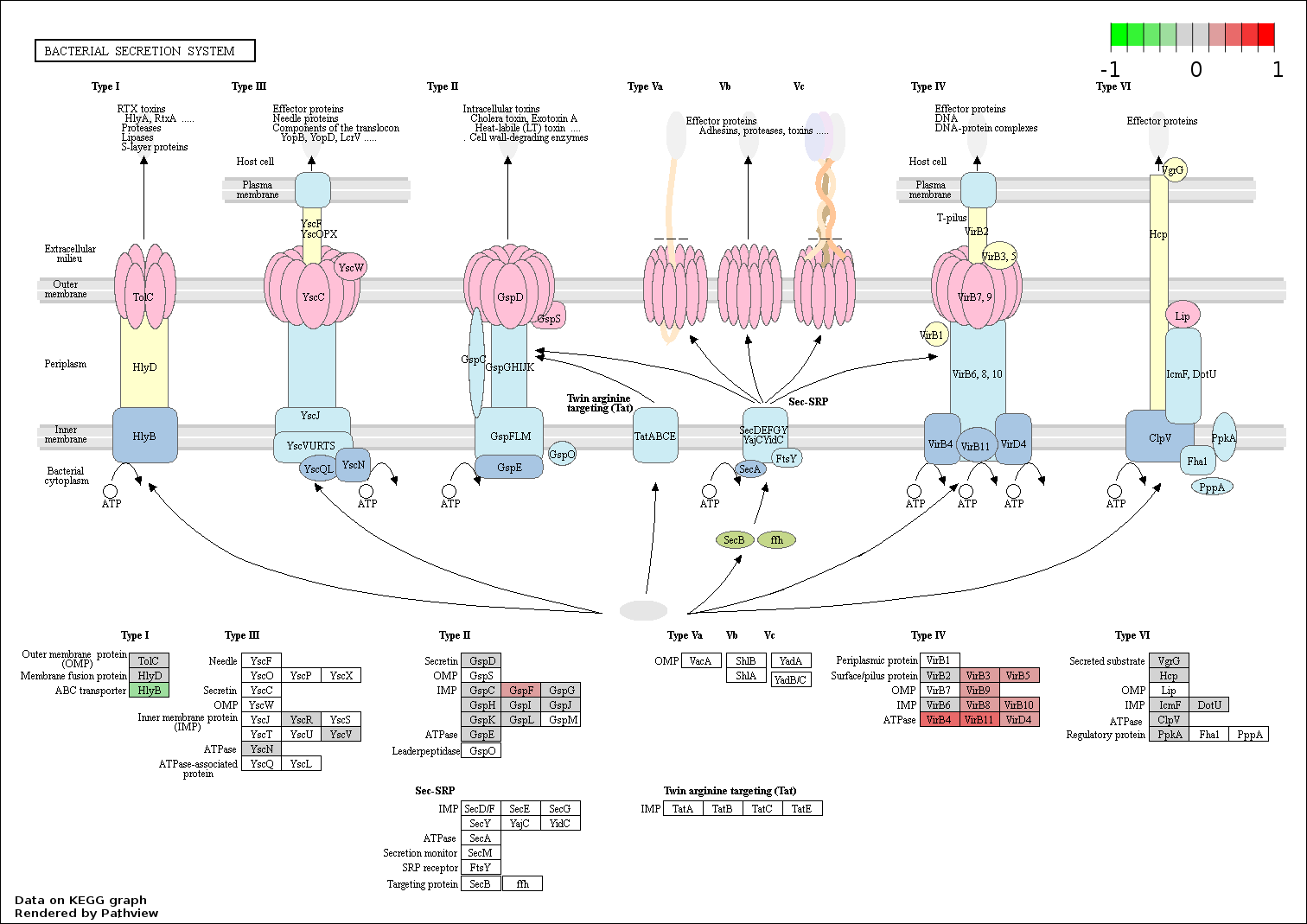


Figure S3 a, b and c: Pathview showing the results of DESeq2 analysis of the category environmental information processing. Red and green represent reads detected in higher or lower abundance in ARD compared with CO, respectively.
